# Ebola viral dynamics in nonhuman primates provides insights into virus immuno-pathogenesis and antiviral strategies

**DOI:** 10.1038/s41467-018-06215-z

**Published:** 2018-10-01

**Authors:** Vincent Madelain, Sylvain Baize, Frédéric Jacquot, Stéphanie Reynard, Alexandra Fizet, Stephane Barron, Caroline Solas, Bruno Lacarelle, Caroline Carbonnelle, France Mentré, Hervé Raoul, Xavier de Lamballerie, Jérémie Guedj

**Affiliations:** 1 IAME, UMR 1137, INSERM, Université Paris Diderot, Sorbonne Paris Cité Paris, 75018, Paris, France; 2 UBIVE, Institut Pasteur, Centre International de Recherche en Infectiologie, 69007 Lyon, France; 3 Laboratoire P4 Inserm-Jean Mérieux, US003 Inserm, 69365 Lyon, France; 4 Aix-Marseille Univ U105, APHM, SMARTc CRCM Inserm UMR1068 CNRS UMR7258, Hôpital La Timone, Laboratoire de Pharmacocinétique et Toxicologie, 13005 Marseille, France; 5 UMR “Emergence des Pathologies Virales” (EPV: Aix-Marseille university - IRD 190 - Inserm 1207 - EHESP) - Institut Hospitalo-Universitaire Méditerranée Infection, 13385 Marseille, France

## Abstract

Despite several clinical trials implemented, no antiviral drug could demonstrate efficacy against Ebola virus. In non-human primates, early initiation of polymerase inhibitors favipiravir and remdesivir improves survival, but whether they could be effective in patients is unknown. Here we analyze the impact of antiviral therapy by using a mathematical model that integrates virological and immunological data of 44 cynomolgus macaques, left untreated or treated with favipiravir. We estimate that favipiravir has a ~50% efficacy in blocking viral production, which results in reducing virus growth and cytokine storm while IFNα reduces cell susceptibility to infection. Simulating the effect of delayed initiations of treatment, our model predicts survival rates of 60% for favipiravir and 100% for remdesivir when treatment is initiated within 3 and 4 days post infection, respectively. These results improve the understanding of Ebola immuno-pathogenesis and can help optimize antiviral evaluation in future outbreaks.

## Abstract (web summary)

Optimization of antiviral therapy can be crucial in the management of Ebola virus outbreaks. Here, Madelain et al. use an integrative mathematical model to correlate the dose and the time of treatment initiation with survival rate, enhanced immune response and viral clearance.

## Introduction

The 2013–2016 Ebola virus disease (EVD) outbreak in West Africa has been the deadliest occurrence of the disease since its discovery in 1976, resulting in 28,616 cases, of which 11,310 were fatal^1^. There is no validated therapeutic protocol against EVD and none of the clinical trials performed during the outbreak, using either small molecules^2,3^, monoclonal antibodies^4^, siRNA^5^ or convalescent plasma^6^ could demonstrate a statistically significant reduction of mortality in EVD.

In absence of cases during inter-epidemic period, NHP models are central to understand virus pathogenesis and to assess the efficacy of treatments against Ebola virus and other related hemorrhagic fever viruses^7,8^. In 2016, our group implemented in cynomolgus macaques a model of EVD which well recapitulates the disease in humans, with virus being detectable at day 3, followed by an exponential increase of virus up to day 7 and death between days 8 and 11^9^. The experimental model was used to assess the efficacy of favipiravir, a broad spectrum RNA polymerase inhibitor^10^, demonstrating that high doses of favipiravir administrated intravenously significantly increased survival rate^11^. Of note, the route of treatment administration may be important, as per os administration of favipiravir led to a lower survival rate^12^. Using another NHP model of EVD, promising results were also obtained by USARMIID with a novel polymerase inhibitor, GS-5734 (remdesivir), showing that the course of the infection could be reversed with 100% survival rate when a dosing regimen of 10 mg kg^−^^1^ QD was initiated 3 days after viral challenge^13^. Although experiments with favipiravir and GS-5734 evidenced that direct antiviral drugs can limit virus replication and increase survival, it is yet unknown how these drugs act in vivo and to what extent they could be useful outside prophylaxis or early post exposure in NHPs and, a fortiori, in humans.

For that purpose, it is critical to get a more detailed understanding of the effect of antiviral treatment on the pathogenesis of EVD, and how it may potentiate the innate and adaptive immune response. Studies performed in EVD patients during the previous outbreaks consistently highlighted the deleterious effect of the inflammatory response on the vital prognosis^14–16^ and in particular the negative correlation between high levels of pro- or anti-inflammatory cytokines at study inclusion (IL6, IL10, IL1β, TNFα, MIP1α, MIP1β, and MCP1) and disease outcome^16,17^. Furthermore global immunosuppression state and altered adaptive responses, as suggested by the high level of T cells expressing inhibitory molecules CTLA-4 and PD-1, were also associated with fatal outcome^16^. In contrast a strong CD8 T cell response was reported in survivor patients^18^, suggesting that the adaptive immune response is key to achieve viral clearance. However, the mechanisms involved in viral clearance are still poorly known, due to the clinical, technical, and ethical difficulties to collect large dataset in humans. Here we collected repeated measures of various markers of the inflammatory and the immune responses during acute infection in 44 untreated and treated cynomolgus macaques^19,20^. Taking advantage of the fact that animals treated with favipiravir had an extended survival while untreated ones died within 11 days, we could describe the dynamics of these markers not only from infection to death but also, in some animals, to viral clearance. This allowed us to assess the relationship between viral replication, inflammatory response and lymphopenia^14–17^, and to explore the role of antiviral treatment in potentiating innate and adaptive immune response in viral clearance^18^.

Mathematical modeling has provided a quantitative understanding of viral dynamics for a number of acute infections, including influenza, dengue or Zika virus^21–24^. In this study we use the techniques of mathematical modeling to analyze the large amount data collected in these animals and in particular to characterize the role of antiviral treatment in potentiating the innate and adaptive immune responses^18^ and improving survival. We discuss the implications of these results to optimize antiviral treatment in future Ebola outbreaks.

## Results

### Survival and virological response

**Fig. 1:**
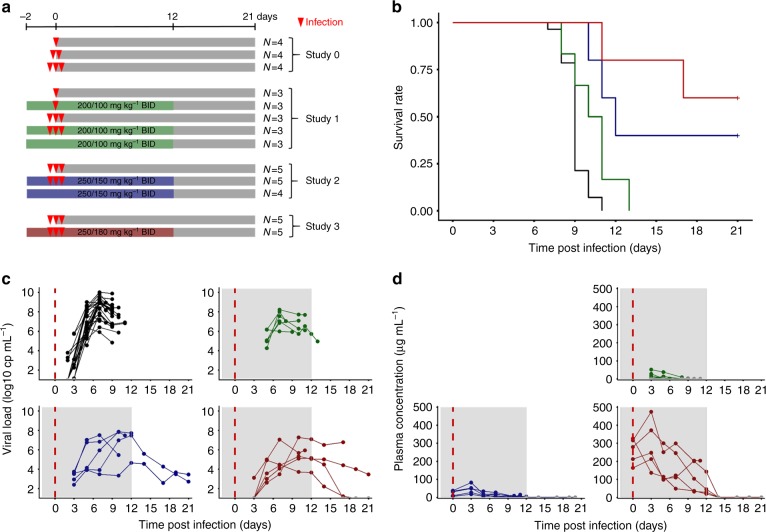
Survival and virological data. **a** Design of the four infected NHP experiments. **b** Survival curves. **c** Ebola virus plasma viral load. **d** Favipiravir plasma concentrations. Dosing regimen groups were untreated (black), 100 mg kg^−1^ BID (green), 150 mg kg^−1^ BID (blue), and 180 mg kg^−1^ BID (red). Gray areas correspond to dosing periods

A total of 44 animals were challenged with Ebola virus Gabon 2001 strain and followed for 21 days over 4 successive experiments (Fig. 1)^9,11^. This included 28 animals untreated and 16 treated with doses of 100, 150, or 180 mg kg^−1^ (*N* = 6, 5, and 5, respectively) of favipiravir given intravenously twice a day (BID) for 14 days, starting 2 days before infection. All animals left untreated died within 11 days post infection. In contrast, increasing doses of favipiravir significantly extend animal survival (*p* value = 0.022; *p* value < 0.001, and *p* value < 0.001 in macaques receiving 100, 150, and 180 mg kg^−^^1^ BID respectively, logrank test), leading to an overall survival rate of 50% (5/10) at day 21 in macaques receiving 150 or 180 mg kg^−1^ (Fig. 1). Survivor macaques at D21 post infection normalized their clinical score and achieved undetectable levels of infectious titers in plasma^11^, as well in liver and spleen (Supplementary Table 1).

### Description of cytokines and CD8 T cell dynamics

**Fig. 2:**
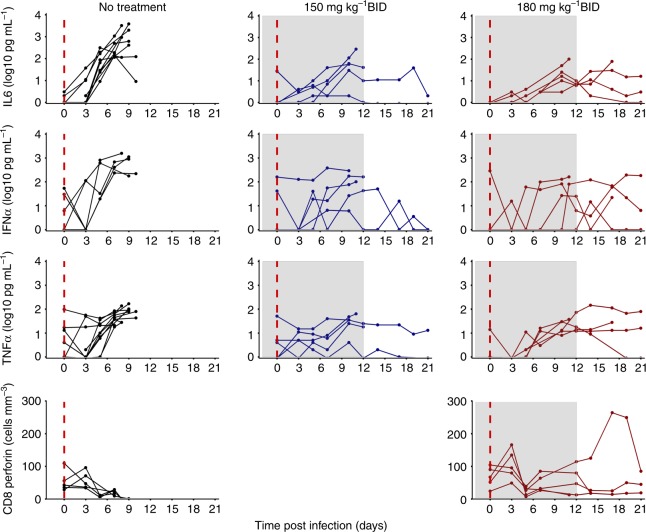
Cytokine and immunological data. Plasma pro inflammatory cytokines Interleukin-6, Interferon α and Tumor necrosis factor α, and CD8 T cells expressing perforin in macaques left untreated (black, left panels), receiving favipiravir 150 mg kg^−1^ BID (blue, middle panels) or 180 mg kg^−1^ (red, right panels). Gray areas correspond to dosing periods

All untreated animals had a cytokine storm characterized by a continuous increase in cytokines levels, in particular pro inflammatory IL6, TNFα, IFNα, from day 5 post infection (D5) to the time to death (Fig. 2). Animals treated with 150 or 180 mg kg^−1^ favipiravir BID showed a delayed peak of cytokine levels and much lower levels of pro-inflammatory than untreated animals, even for those that did not survive up to D21.

In the subset of 10 animals where these data were available, a profound lymphopenia was observed in all lymphocyte subpopulation, with a nadir time occurring between D7 and D11, with median [min–max] value of 511 [49–993] cells per mm^3^, consistent with what is observed during EVD^15,20^. In the 4 animals that had an extended survival, CD4 and CD8 T cell counts rapidly increased after D10, in particular those expressing cytotoxic surface markers (granzyme B, perforin), activation surface markers (CD95 + ) and memory markers (CD27-, CD28-, CD45RA-) (Fig. 2 and Supplementary Figure 1).

### Association between cytokine levels and disease progression

At D7, viremia significantly correlated with a number of cytokines, in particular IFNα (*r* = 0.89, *q* value < 0.001, Spearman correlation test), and IL6, IFNγ, MIP1β, MCP1, G-CSF (all *q* values < 0.05, Spearman correlation test) to a lesser extent (Supplementary Table 2). The association between levels of inflammation at day 7 and survival times was even greater, with a large number of cytokines having a significantly negative correlation with survival times (Supplementary Table 2, Supplementary Figure 2). The largest association between cytokine value at D7 and survival time was found for IL6 (*r* = −0.93, *q* value < 10^−5^, Spearman correlation test), consistent with clinical observations^17^, and this correlation was larger than that between viremia and survival (*r* = 0.79, *p* value = 3.4×10^−5^, Spearman correlation test). Overall six cytokines (IL6, IFNα, G-CSF, IL10, IL1RA, and IFNγ) had a higher correlation rate with survival time than viremia (Supplementary Table 2).

### Integrated model of viral and immune response dynamics

Mathematical models of increasing complexity were used to fit EBOV viremia in untreated and treated animals (Supplementary Table 3). All models shared a number of assumptions, in particular the facts that (i) blood compartment was a good reflect of infection, (ii) a single compartment was used for the target cell populations, (iii) infected cells went through an eclipse phase before being productively infected, and the duration of this eclipse phase was exponentially distributed, (iv) favipiravir reduced viral production in a concentration dependent manner (detailed in Supplementary Methods). The model focused on the systemic infection, relying on measurement in blood, and did not include specific immune preserved sites such as genital tract or eyes where the kinetic of the infection may be different. As the virus has a broad cell tropism and disseminates early in the infection in blood and lymph circulation, the model assumes that the various cellular types targeted by the virus (including monocytes, hepatocytes, adrenocortical cells, fibroblast, and epithelial cells^25^) can be summarized into one target cell population, with homogenous repartition in the body.

The first model was a standard target cell limited model, which predicts that peak viremia occurs when the pool of susceptible cells has been largely depleted^21^. Although the model could provide a good fit to each individual viremia, model based predictions showed that it under-predicted the effect of high doses of treatment (Supplementary Methods), suggesting that the exhaustion of susceptible cells was not sufficient to explain viral dynamics.

Next, we analyzed whether viral load description could be improved by taking into account the effects of the innate immune response on viral replication (Supplementary Methods). We assumed that the production of type I IFN by activated macrophages^26^ was proportional to the number of infected cells. We tested in the model the main mechanisms by which the upregulation of IFN stimulated genes^26^ impacted viral replication, namely increasing cell refractoriness to infection^27,28^, reducing viral replication from infected cells^28,29^, increasing the loss rate of infected cells^29,30^ or increasing target cell availability^31,32^ (see Methods). Models assuming that pro-inflammatory cytokines increased cell refractoriness to infection consistently provided the best fit to the viral load, allowing to capture the dose effect relationship on viremia (Supplementary Methods). Given their high level of correlations, a similarly good prediction of the viremia was obtained when assuming that this effect was driven by either IFNα, IL6, or TNFα. Because the effects of IFNα is supported by in vitro experiments^27,33^, we decided in the following to include only the effect of IFNα; given the high level of correlation and the variability in cytokine dynamics, we kept IL6 and TNFα in the model as instrumental variables reflecting the overall level of cytokine response.

**Fig. 3:**
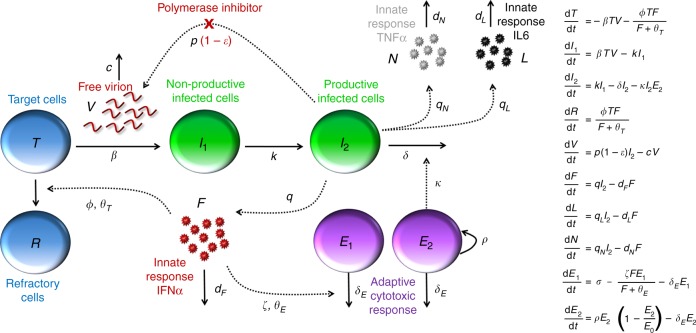
Schematic and mathematical model of Ebola virus infection. As the virus early disseminate in blood and lymph circulation, the model assumes only one target cell compartment. After infection by free virus (*V*), target cells (*T*) enter an eclipse phase (*I*_1_) before becoming productively infected (*I*_2_), which activate macrophage and production of cytokines. Cytokine release, in particular IFNα, leads to an increase of cells that are refractory to infection (*R*), and triggers apoptosis of non-specific CD8 T cells (*E*_1_), which creates room for proliferation of EBOV-specific CD8 T cells (*E*_2_) that eliminate productively infected cells *I*_2_ Polymerase inhibitors as favipiravir inhibit viral production with efficacy ε

Thirdly, the model was extended to include the adaptive response, assuming a decline of non-specific cells and an expansion of specific cytotoxic cells. Including CD8 T cells expressing perforin provided the best improvement of the viremia data description and could reproduce both the cytokine-mediated lymphopenia observed in early infection and the rapid viral decline in NHPs after peak viremia. The ordinary differential equation system and a schematic representation of the final selected viral dynamic model are given in Fig. 3. The corresponding code to estimate the model parameters and performe simulations is provided in Supplementary Software.

### Impact of viral and cytokine dynamics on disease progression

**Fig. 4:**
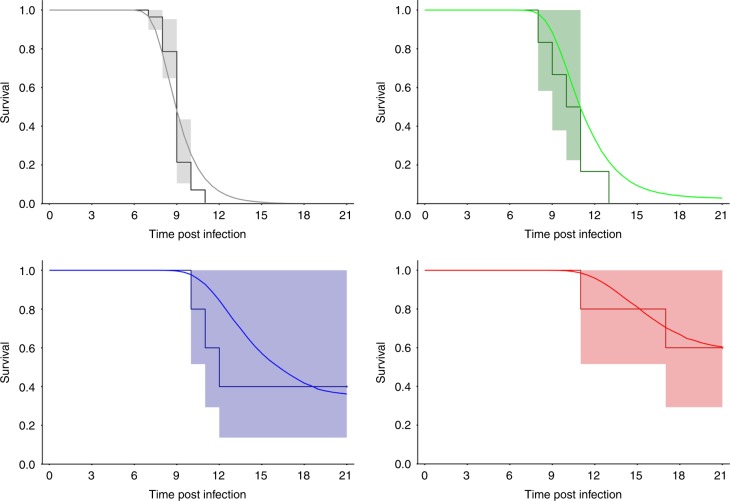
Model-based predicted survival rate (plain lines) and Kaplan–Meier survival curves and 95% confidence interval (shaded areas) for each dosing regimen group. Top left: cynomolgus experiment control pooled from the 4 experiments (*N* = 28), top right: favipiravir 100 mg kg^−1^ BID (*N* = 6), bottom left: favipiravir 150 mg kg^−1^ BID (*N* = 5), bottom right: favipiravir 180 mg kg^−1^ BID (*N* = 5). In each panel, model-based predicted survival rate was calculated by simulating 1000 individuals for each dosing regimen and taking the medians of the predicted individual survival functions at each time

Next, we investigated the impact of viral and cytokine dynamics on survival times. Tissue damage in EBOV-infected individuals is caused by direct viral-induced cytopathic effects and indirect organ injury mediated by host inflammatory responses, endothelial dysfunction, and disordered coagulation^34^. Activation of the monocytes/macrophages induces the release of multiple pro inflammatory mediators, including (TNF)-α, interleukin (IL)-1, IL-6, and nitric oxide, which induces cell apoptosis or necrosis^35^. Thus in our model, we assumed that viral load (including a lag-effect) could impair the instantaneous risk of death. We found that this model provided a less good description of the distribution of time to death than a model assuming that it was impacted by either one of IL6, TNFα or IFNα (including a lag-effect). Only the effect of IFNα on survival was kept in the model, which well recapitulated the mortality rate observed until D21 in all dosing group regimens (Fig. 4). The lag effect constant was estimated to 0.3 per day corresponding to an average delay of 3.1 days to impact NHP survival.

### Viral dynamic model predictions and parameters

**Fig. 5:**
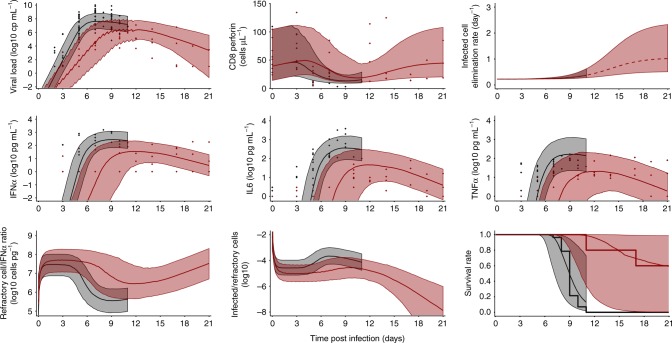
Individual observation (dots) and median model predictions (line) in animals left untreated (black) or treated with 180 mg kg^−1^ BID favipiravir (red). Top left: Ebola virus viral load; top middle CD8 T cells expressing perforin; top right: model prediction of productive infected cell elimination rate, increasing concomitantly to adaptive response; middle left: IFNα concentrations; middle: IL6 concentration; middle right: TNFα concentration; bottom left: predicted ratio of protected cells on the concentration of IFNα (*R*/*F*); bottom middle: model prediction of the ratio of infected on protected cells (*R*/*I*_2_); bottom right: Kaplan Meier curves and survival rate prediction. Shaded areas represent the predictions within the 10th and 90th percentiles of 1000 simulations

In acute infection, peak viremia occurs when the number of newly infected cells does no longer compensate for the loss of infected cells. In our integrated model the main cause of reduction in cell infection was not due to the depletion of target cells (like in the target cell limited model) but to the fact that IFNα increases the number of target cells that are refractory to the infection. In fact, the model predicted that low levels of IFNα of about 1.7 pg mL^−1^ (Table 1) are sufficient to induce half of the maximal conversion rate of susceptible cells to an antiviral state. However, IFNα concentrations greater than about 17 pg mL^−1^ provide very little additional benefit for cell protection. Thus, in untreated animals the infection leads to a massive release of IFNα (with median levels of 400 pg mL^−1^ at peak viremia, Fig. 5) that negatively affects survival rate while having only little effect on limiting cell infection. On the contrary, in treated animals, favipiravir reduces viral production, and hence the number of infected cells and IFNα concentrations are lower. This is sufficient to confer a nearly similar effect on cell infection, reduce and delay peak viremia while limiting the deleterious effect of cytokine storm on survival (Fig. 5). We also verified that including target cell regeneration did not modify the model predictions, assuming a proliferation rate value no greater than 1 per day.

**Table 1:** Parameter estimates of the final viro-immunologic model of Ebola virus disease in cynomolgus macaques

Parameter	Name	Unit	Fixed effect		sd of the random effect	
			Estimate	r.s.e.(%)	Estimate	r.s.e.(%)
Baseline clearance rate of productively infected cells	*δ*	Per day	0.224	9	0.169	40
Viral production	*p*	Virion per cell per day	4.15×10^4^	71	1.76	18
Clearance rate of virions	*c*	Per day	20	–	0	–
Initial number of target cells	*T*_0_	Cells per mL	10^8^	–	0	–
Inoculum size	*V*_0_	Virion per mL	10^−4.1^	11	1.5	17
Virion infectivity	*β*	mL per virion per day	7.9×10^−11^	73	0	–
Rate of transition to productively infected cells	*k*	Per day	4	–	0	–
Favipiravir maximal effect	*E*_max_		1	–	0	–
Drug concentration giving 50% of maximal effect	EC_50_	µg per mL	191	20	0	–
IFNα production rate	*q*	pg per cell per day	0.0074	69	0	–
IFNα elimination rate	*d*_F_	Per day	0.4	–	0	–
IFNα concentration giving 50% of max effect	*θ*_T_	pg per mL	1.73	35	1.05	16
Maximal rate of transition from target to refractory cells	*ϕ*	mL pg per day	2.67	17	0	–
CD8 T cell perforin+ baseline value	*C*_0_	Cell per mL	36,900	30	0.775	23
Initial proportion of EBOV-specific CD8 T cell perforin+	*P*_0_		0.001	–	0	–
CD8 T cell perforin+ elimination rate	*δ*_E_	Per day	0.001	–	0	–
CD8 T cell perforin+ elimination rate mediated by viremia	*ζ*	mL per virion per day	0.455	41	0	–
Specific CD8 T cell perforin+ growth constant	*ρ*	Per day	0.338	11	0	–
Non specific CD8 T cell perforin+ growth constant	*σ*	cell per mL per day	3050	87	0	–
IFNα concentration providing 50% of max effect on CD8 T cell perforin+ depletion	*θ*_E_	pg per mL	6.5×10^−4^	233	0	–
CD8 T cell perforin+ mediated infected cell elimination rate	*κ*	Per day per cell per mL	2.08×10^−5^	31	0	–
IL6 production rate	*q*_L_	pg per cell day	0.0097	65	0.76	37
IL6 elimination rate	*d*_L_	Per day	0.4	–	0	–
TNFα production rate	*q*_N_	pg per cell per day	0.0046	69	1.03	30
TNFα elimination rate	*d*_N_	Per day	0.4	–	0	–
Maximal hazard of death	*λ*_m_	Per day	1.12	2	0	–
IFNα effect compartment concentration inducing 50% of the maximal hazard	*F*_50_	pg per mL	103	12	0	–
Transfer constant	*k*_s_	Per day	0.319	3	0	–
Hill coefficient	*γ*		2	–	–	–

Parameter of the longitudinal and the joint models were sequentially estimated

sd: standard deviation, r.s.e.: relative standard error

### Impact of CD8 T cell dynamics on infected cell half-life

The main effect of the specific adaptive response in the model was to increase the clearance rate of productively infected cells, and this was supported by the fit of the CD8 T cells expressing cytotoxicity surface markers. In the days that follow infection, the half-life of infected cells was estimated to about 3 days (*δ* = 0.22 per day), suggesting that in absence of adaptive response, it would take several weeks to clear viremia. However the loss rate of infected cells increased with the levels of CD8 T cell, in particular those expressing perforin, leading to a much shorter half-life of about 16 h (*δ* = 1 per day) at D21 in animals that survived, which explains the rapid clearance of viremia observed after peak in surviving animals (Fig. 5).

### Antiviral effectiveness of favipiravir

Given the pharmacokinetics of the drug (see Supplementary Methods), one can estimate the in vivo drug EC_50_, equal to 191 µg mL^−1^ (Table 1). Importantly this estimate was robust in all the viral kinetic models considered and in the sensitivity analysis (Supplementary Table 4, Supplementary Methods). Accordingly, the effect of favipiravir in impairing viral replication was modest with maintenance doses of 150 and 180 mg kg^−1^ BID leading to a median effectiveness of 40 and 50%, respectively. Simulation suggested that higher doses (250 and 300 mg kg^−1^ BID) may increase median effectiveness to 66 and 72% respectively, and survival rate at day 21 to about 80% (Supplementary Figure 3).

### Validation of the model on rhesus macaques receiving GS-5734

We assessed whether the mathematical model could be applied to a different animal model and another antiviral drug, namely GS-5734, a potent nucleotide polymerase inhibitor. In this experiment from literature^13^, 12 rhesus macaques were challenged by 1000 focus forming unit (ffu) of EBOV and 6 animals were treated with 10 mg kg^−1^ QD of GS-5734 initiated at D3 post infection (see Methods and Supplementary Figure 4)^13^. The drug antiviral effectiveness was estimated to 88%, and this could well fit the viral load data observed in all animals, in particular the sharp reduction in viral levels after treatment initiation (Supplementary Figure 4). With this level of efficacy, the model well reproduced the survival rates of 0 and 100% observed in untreated and treated animals, respectively, showing that the model could be relevant to predict survival in a different experimental setting.

### Impact of drug efficacy and timing of initiation on survival

**Fig. 6:**
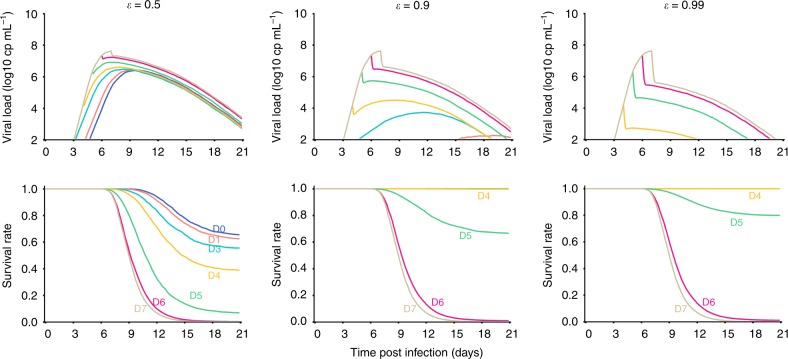
Viral load and survival rate predictions for various levels of drug efficacy and timing of treatment initiation, assuming no pharmacokinetic variability. Left: *ε* = 50% (eg, favipiravir), middle: *ε* = 90% (eg, GS-5734); right: *ε* = 99%. Dark blue: treatment initiation at D0, magenta: D2, light blue: D3, yellow: D4, green: D5, brown: D7. Results show the median of 1000 simulations

Next we used the model to predict the impact of various levels of efficacy and timing of treatment initiation on survival. For that purpose we neglected PK related variations and assumed constant drug effectiveness of 50 and 90%, respectively, which correspond to the median efficacy observed with favipiravir 180 mg kg^−1^ BID and GS-5734 10 mg kg^−1^ QD, respectively. In the case of favipiravir, delaying treatment initiation up to 3 days after viral challenge was predicted to marginally affect survival, with a survival rate between 60 and 70% in all cases (Fig. 6). However treatment initiated at D5 or after led to a survival rate of less than 10%. Treatment with a more potent drug such as GS-5734 could achieve 100% survival if initiated to D4 and 70% survival if initiated to D5. Yet, treatment initiated at D6 or after led to a predicted survival rate of 0%.

Given these levels of efficacy, the combination of the two drugs may only slightly improve survival compared to GS-5734 alone, with a combined drug effectiveness of 95% (Supplementary Figure 5). However, associating favipiravir with another drug having a similar potency would result in a combined effectiveness of 75%. This may allow to achieve 100% survival if treatment is initiated up to D3 and 90% if treatment is initiated up to D4 (Supplementary Figure 6).

In order to achieve 100% survival up with a treatment initiated at D5, we estimated that drug antiviral effectiveness would need to be larger than 99%, i.e., larger than what is achieved with GS-5734 alone or in combination with favipiravir. However, despite reducing viral load levels, such level of high effectiveness would yet not be sufficient to reverse the course of the disease if treatment is initiated after D5 (0% survival). This reinforces the idea that, in order to be effective, a purely antiviral treatment needs to be administered at least 2 days before peak viremia and cytokine storm (Fig. 6). However, simulations assuming other mechanism of action of the drug, such as increasing the loss rate of infected cells, could extend the time window of intervention (Supplementary Figure 7).

## Discussion

This study provided an integrated picture of EVD pathogenesis and the role of viral polymerase inhibitors to avert disease progression. The systematic approach used to construct our model showed that the best description to the data was obtained assuming that the main role of the innate response during early infection was to increase the number of target cells refractory to the infection, while the cytotoxic adaptive response, in particular the CD8 T cells expressing perforin, significantly increased the elimination rate of infected cells after peak viremia (D7). In this interplay between the host and the virus, favipiravir inhibits viral production from infected cells, with an efficacy close to 50% at the highest doses used, allowing to delay the exponential growth of infected cells and the deleterious effect of cytokine storm on survival. This virtuous interaction between treatment and innate immune response explains our prediction that about 60% survival could obtained if treatment is given in the first 3 days of infection (Fig. 5).

The cytokine dynamics were largely consistent across animals, with uncontrolled levels of cytokines in untreated animals, strongly associated with time to death and reflecting the shock state leading to multi-organ failure and death observed in these animals^36^. In contrast, animals treated with 150 or 180 mg kg^−1^ BID favipiravir exhibited much lower levels of pro-inflammatory cytokines than untreated animals. By comparing the dynamics in animals that survived and those that did not survive, the model proposes a quantitative understanding of the antagonist effects of the inflammatory response observed in our data here and elsewhere^14,15^. The model predicts that low concentrations of circulating IFNα of only 1.7 pg mL^−1^ are sufficient to induce half of the maximal conversion rate of target cells to refractory cells. This is much lower than the values observed in untreated animals, which increased up to 400 pg mL^−1^. Thus in untreated animals large amount of IFNα are released that negatively affects survival rate without increasing much the level of immunity. This is consistent with the dual roles played by type I IFN during infections. Indeed, besides their beneficial direct antiviral effect, type I IFN may be deleterious when released at high levels and for a prolonged period. These effects include increase of inflammatory mediator release, depletion of T cells, destruction of the secondary lymphoid architecture, and inhibition of hematopoiesis, all of them likely occurring also during severe Ebola virus disease in humans^37,38^. Reversely, treatment with favipiravir reduces viral replication, which downregulates IFNα and other cytokines release and confers a nearly similar level of cell protection while limiting the deleterious effect of the inflammatory response. This allows the animals to pass the peak viremia and to give time for the induction of an effective adaptive immune response after D7. Consistent with our assumption on IFNα limiting cell infection, we previously reported that EBOV viral dynamics in type I IFN receptor knock out mice could be captured by a simple target cell limited model ignoring the impact of the innate immune response^39^. Further, untreated mice had an extensive hepatic cytolysis^40^ while the levels of ALT observed here were much lower (median peak value < 500 IU L^−1^), supporting the hypothesis that the innate response limits cell depletion in NHP.

The adaptive CD8 T cell response could only be observed in animals that had an extended survival, which were all treated with 180 mg kg^−1^ BID. Including the effect of the CD8 T cell response in reducing infected cell half-life significantly improved data fitting. In the days that follow infection, the half-life of infected cells was estimated to about 3 days, suggesting that in absence of an adaptive response, it would take several weeks to clear viremia. However, the increase in the cytotoxic adaptive response led to a rapid decrease of the infected cell half-life (16 h at D21), allowing viral clearance to occur in about 10 days after the peak, at least in peripheral blood. These results, albeit obtained on a limited number of animals, are consistent with the description of a strong cellular response in survivor patients^18^ and with the potential role played by this response in the control of EBOV infection. Although the model used for the adaptive immune response could well reproduce the limited body of data available, more complex models may be needed in the future to take into account other factors such as T-cell redistribution or bystander activation of non-antigen specific T-cells^41^.

Our analysis therefore supports the following chain of events: exponential viral replication during acute infection leads to a rapid increase in cytokine levels, which in turn limits cell infection and viral spread but also increase lymphopenia and inflammation with direct impact on vital prognosis. The introduction of an antiviral drug in prophylaxis or post exposure, even with a moderate effectiveness, impairs the viral replication, and thus limits the pro-inflammatory cytokines production, sparing time to induce an effective adaptive response and finally improves survival. Therefore, favipiravir allows the induction of adaptive immunity but also decreases the pathogenesis due to cytokine storm. Our estimate of favipiravir EC_50_ against EBOV is about 191 µg mL^−1^, which, assuming a binding fraction of 50%^42^, would correspond to a free concentration of 96 µg mL^−1^. This value is above the IC_50_ reported in the literature, ranging from 10.5 to 62 µg mL^−1^ ^40,43,44^, and explains why the group treated with 100 mg kg^−1^ BID, targeting trough concentrations close to the IC_50_, did not show strong antiviral efficacy. Simulations predict that dosing increase to 250 or 300 mg kg^−1^ BID may allow to reach average efficacy *ε* of about 66 and 73% respectively (Supplementary Figure 3) but toxicity issues at these dosing regimens have been reported^45^.

Using the model to simulate various antiviral strategies we found that delaying initiation of favipiravir up to 3 days post infection should not have a major impact on survival rate. Applying the model to the dynamics observed during treatment with the polymerase inhibitor GS-5734^13^ predicted 100% survival if treatment is initiated up to 4 days post infection, but not after. As survival appears to be directly related to the level of the cytokine storm, antiviral treatment, even if strongly effective, needs therefore to be administered at least 2 days before viral peak to be effective (Fig. 6). In the future it will be important to see if favipiravir initiated few days after viral challenge in NHPs could provide survival rate matching with the model predictions. Whether this model can be applied to other therapeutic classes, in particular monoclonal antibodies such as ZMapp^4^, is another question to be addressed. Assuming that monoclonal antibodies act mainly by reducing the lifespan of infected cells, we estimated that a reduction of the lifespan by a factor 5 could reproduce the high survival rate observed in NHP experiments^4^, even if initiated until day 5 or day 6 post infection (Supplementary Figure 7). In spite of the large amount of data available, our model made a number of hypotheses. First, some parameters had to be fixed to grant identifiability. Although the sensitivity analysis showed that the model predictions were robust to changes in the fixed parameters (Supplementary Table 4), more data on the phenotype of infected cells will be needed to improve the understanding of EBOV pathogenesis. Second, as done in previous works^46,47^, we relied on total concentration measurement of favipiravir as the driver of antiviral efficacy, an assumption supported by the short half-life of intracellular active favipiravir ribosyl triphosphate^44,48^.

Obviously, the translation of results from NHP experiments to patients needs to be done cautiously. First, unlike what is obtained in the NHP model, EVD is not uniformly lethal and mortality rate during the last outbreak is close to 40%^49^. Thus, it is possible that our model is more stringent and that treatment initiation of a potent drug, close to peak viremia, may nonetheless have an impact on the disease and the survival, when infection is made with lower inoculum and supportive care is provided. In the last outbreak, the time from symptom onset to admission was between 3 and 5 days^2,4,50^, when the maximal viral load is 4–5 days after symptom onset^50^. Based on these observations, it is likely that most patients included in EVD clinical trials, such as JIKI (favipiravir) or Prevail (ZMapp) initiated therapeutics close to the viremia peak and several days after virus replicated at high levels^2,4^. Consistent with our prediction that antiviral drugs should be initiated as early as possible to reach maximal therapeutic benefit, the effect on survival in these studies was modest for favipiravir^47^, and stronger but yet not statistically significant for ZMapp^4^. Even in the case of a more potent drug such as GS-5734, our results suggest that the efficacy may also be largely contingent on the timing of treatment initiation. In the future, it will thus remain critical to administrate drugs not only to confirmed or suspect cases, but also to contact individuals as early as possible, in a context where the NHP model demonstrated their efficacy. This may meet the wish of field teams during the Western African EVD outbreak to propose an early oral treatment to suspected cases, even before their transfer to care centers. Antiviral strategy may also offer an alternative to vaccine, or be complementary to it, for exposed people, as the vesicular stomatitis virus-based vaccine provide high level protection after a 10 days delay, but its efficacy in post exposure in both humans and NHPs is less clear^51,52^. Interestingly, as written in^53^ favipiravir has been used as post-exposure prophylaxis in at least five health-care workers with percutaneous accidents and suspected Ebola virus exposures during the west Africa outbreak. None of these individuals developed laboratory or clinical evidence of Ebola virus infection, but whether any infections were prevented by the use of post-exposure prophylaxis, as supported by our model, is not possible to determine from this small number of uncontrolled cases.

In conclusion, we proposed a mechanistic mathematical model to assess the virological and immunological dynamics of EBOV infection in NHPs and to assess the efficacy of favipiravir in reducing virus pathogenesis. Applying the model to different levels of antiviral strategies with various efficacy and delay of initiation, our simulations predict that in order to be effective, antiviral treatment should be given at least one or 2 days before peak viremia. These results support a window to preventive or post exposure therapeutic strategies, with the aim to define what should be the drugs and the dosing to evaluate in case of new EVD outbreak.

## Methods

### Description of the experiments

We used data of four successive experiments performed in the Inserm-Jean Mérieux biosafety level 4 laboratory in Lyon in cynomolgus macaques that were left untreated or were treated by intravenous favipiravir (Fig. 1a, Supplementary Table 3). Briefly, female cynomolgus macaques from Mauritius (3 years aged and weighting 2.8-4 kg) were challenged intramuscularly with dose inoculum of 10, 100, or 1000 focus forming units (ffu) of Ebola virus Gabon 2001 strain, with no difference in survival or viral kinetics across the different inoculum groups^9^. Zaire Ebola virus Gabon 2001 strain was chosen due to its high observed lethality in patients and its availability at the beginning of the experiments. Treatment was initiated 2 days before infection, and favipiravir was administered twice a day every 12 h, by 10 min infusion, after intramuscular anesthesia using Zoletil (Tiletamine/Zolazepam). Overall, the data of 44 animals were analyzed, 28 left untreated and 16 treated with maintenance doses of 100, 150, and 180 mg kg^−1^ BID (*N* = 6, 5, and 5, respectively). Animals were euthanized at the latest at D21 (study endpoint) but moribund animals were euthanized before to alleviate unnecessary suffering. All untreated animals died within 11 days of infection, and study endpoint was achieved in 0, 2, and 3 animals receiving 100, 150, and 180 mg kg^−1^ BID, respectively (i.e., 0, 40, and 60%, respectively). Animals were housed and monitored in accordance with the guidelines of the European directive 2010/63 and procedures established for use of animals in BSL4 facilities. Protocols and experiments received ethical authorizations, number P4-2014-008, 2017APAFIS#6097, and 2016062713281115 from the CECCAPP C2EA15 ethical committee, registered with the French Ministry of Research. The design of the experiments has been described in detail elsewhere^9,11^.

### Data collected

Blood samples were collected at days 0, 2 or 3, 5, 7, 9, or 10, 12, 14, 17, 19, and 21 post infection, within 15 min before of the first administration of favipiravir of the day. EBOV plasma molecular viral load (Fig. 1) was measured by real time PCR using the Gibbs system^9^. Plasma favipiravir concentrations (Fig. 1) were assessed using HPLC coupled to UV detector following a previously validated procedure^47^, with a limit of quantification of 1 µg mL^−1^.

Repeated measurements of immunological markers were assayed in treated macaques and control macaques of the corresponding experiment. A panel of 37 plasma cytokines, chemokines, growth factors, chemotactic factors were assayed using Luminex technology, Magpix instrument, at days 0, 3, 5, 7, 10, 12, 14, 17, and 21 post infection, and on day of euthanasia, in treated (*N* = 10) and untreated (*N* = 10) animals of the third and fourth experiments evaluating the doses of favipiravir of 150 and 180 mg kg^−1^ BID, respectively (Supplementary Table 2). The panel included IL1β, IL1RA, IL2, IL4, IL6, IL8, IL10, IL15, IL18, IL23, eotaxin, fractalkine, MCP1, MIP1a, MIP1β, CXCL10, IFNγ, TNFα, TNFβ, FGFb, G-CSF, GM-CSF, VEGF, sCD40L, sCD95L, and sCD137. In addition, interferon α (IFNα) plasma concentration was assayed using ELISA method in the same animals, however data from untreated animals in the study 3 could not be obtained (hence these data were obtained in *N* = 15 animals). Second, the distribution of lymphocyte populations was explored in the animals of experiment 3 (*N* = 10) with the expression or absence of expression of 12 biomarkers reflecting activation status, cytotoxic activity and memory phenotype: HLADR, KI67, granzyme B, perforin, CD154, CD69, CD152, CD45RA, CD27, CD28, CD95, CD137, and NKp80. Of note among these 10 animals, 5 were untreated and died within 10 days (Fig. 1), 4 treated animal had an extended survival while 1 treated animal died at D11.

Viral loads in liver and spleen homogenates (after grinding of 30 mg) were measured by plaque assay. Standard 12 well microplates of VeroE6 cells were prepared one day before titration. Cells were infected with serial dilutions of samples during 1 h at 37 °C. After incubation, 1.5 ml of CMC per well were added and incubated at 37 °C for 7 days. Titres were determined by immunohistochemistry after staining with a specific antibody.

### Descriptive analysis of immunological measurements

Associations between cytokine value at D7 and (i) viremia at D7 (ii) death time were assessed using Spearman correlation test, with *p* values adjusted (*q* values, Hochberg Benjamini method). Seven cytokines showed no increase greater than 25 pg mL^−1^ at peak in more than 50% of the animals in each group (IL1β, IL16, IL23, eotaxin, fractalkine, TNFβ, and soluble Fas ligand) and were excluded from the analysis.

### Favipiravir pharmacokinetics

Favipiravir pharmacokinetics in macaques was described using a model developed previously in uninfected animals^46^. The model was used to fit PK data, to predict the drug concentrations over time and to assess a potential effect of infection on pharmacokinetic parameters (Supplementary Methods, Supplementary Table 5, Supplementary Figures 8, 9 and 10).

### Modeling the interplay between EBOV and the immune response

We aimed to build a comprehensive model of the host-pathogen-drug interaction during EBOV infection. For that purpose, we developed a model of progressive complexity in a four stages strategy (detailed in Supplementary Methods). Of note the nonlinear mixed effect model approach (see below) allowed us to incorporate data collected in all animals at each stage of the analysis, even in case of missing data. In order to ensure model comparability, the different models were compared on their ability to fit the viremia data only.

In the first stage viral dynamics was characterized assuming a standard target cell limited model with an eclipse phase^21^. Favipiravir is a puric basis analog, with several potential effects hampering the RNA virus replication. The most characterized was the inhibition of the RNA polymerase, it blocks the production of new viral genomes and hence the production of new viral particles^48^. The effect of this polymerase inhibitor was assumed to inhibit viral production in a concentration dependent manner as: $\varepsilon = \frac{{E_{\rm max} \times C}}{{\rm EC_{50} + C}}$ where ε is potency of the drug, *E*_max_ the maximal effect, EC_50_ the concentration providing 50% of the maximal effect and *C* the plasma concentration of favipiravir (Supplementary Methods, Supplementary Table 6). There was no difference in survival or viral loads at the different time points across the different inoculum groups^11^. Thus, we assumed that the initial viral load was proportional to the size of the inoculum, and we noted *V*_0_ the normalized initial viral load concentration (with a reference inoculum dose of 1000 ffu), such that the initial viral load was on average 10 and 100 folds lower in the 100 ffu group and the 10 ffu group respectively.”

In the second stage the model was extended to incorporate the effects of the innate immune response, taking the cytokine levels from D0 to D21 as a marker of the innate response (Supplementary Tables 7, 8 and 9, Supplementary Figure 11). Four putative models were considered based on literature results^22,54,55^, assuming that cytokine release could either (i) increase the number of cells refractory to infection^27,28^, (ii) increase the availability of target cells^31,32^, (iii) decrease viral production^28,29^, (iv) increase the clearance of infected cells^29,30^. To limit the number of models to test, we focused only on pro-inflammatory cytokines and cytokines related to cellular response (IFNα, IL6, TNFα, IL2, IFNγ, IL15, IL18, and perforin) that were significantly associated with survival time in the descriptive analysis (Supplementary Table 2).

In the third stage, we evaluated the role of T-cell populations. Based on the descriptive analysis result and given the limited number of data, we focused only on the cytotoxic response and CD8 T cell populations expressing cytotoxic surface markers, i.e., perforin, granzyme B and NKp80 from D0 to D21 (Supplementary Tables 10, 11). For each of these populations the dynamics was modeled assuming one compartment of nonspecific cells having a cytokine-driven apoptosis and one compartment of specific cells that increased over time and eliminate infected cells.

### Modeling the effect of viral and cytokine dynamics on survival

The last step of the model aimed to incorporate the impact of viral and cytokine dynamics on time to death, an approach called joint modeling in the statistical literature^56^. For each animal we note T the time of death and we assumed that the instantaneous risk of death, noted *h(t)*, was defined by $h( t ) = \lambda _{\rm m} \times \frac{{X_{\mathrm k}^\gamma (t)}}{{X_{\mathrm k}^\gamma \left( t \right) + X_{50}^\gamma }}$ where *X*_k_*(t)* is the current or delayed (using an effect compartment) values of viral load or cytokine predicted by the model, *λ*_m_ is the maximal hazard in presence of infection, *X*_50_ is the current or lag- value of viral load or cytokine inducing hazard value equal to 50% of the maximal hazard, and *γ* the Hill coefficient. The probability to survive up to time *t* can then be reconstructed and $S( t ) = {{P}}\left( {{{T}} \ > \ {{t}}} \right) = {\mathrm e}^{ - \mathop {\int }\limits_0^t h( u ){\mathrm d}u}$. The model variable included in the hazard function was selected using the Bayesian information criterion (BIC, the lower the better) (Supplementary Tables 12, 13).

### Parameter estimation

All model estimations were performed using non-linear mixed effect models and the SAEM algorithm implemented in Monolix software (http://lixoft.com), an approach that borrows strengths from the inter-individual variability to increase the precision of parameter estimation^57^. Model of increasing complexity were kept only if they improved the description of the viral load data, and the most parsimonious model was selected at each stage using the log-likelihood of the viremia data (Supplementary Figures 12, 13, and 14). Random effect selection was performed after the best model was selected, using a backward procedure. Model evaluation was performed for the final model using individuals fits (Supplementary Figure 15) and visual predictive check per dose (Supplementary Figure 16)^58^. Time to event model was evaluated using Cox-Snell residuals (Supplementary Figure 17)^59^.

To ensure model practical identifiability^24,60^ the following parameters were fixed in all models: the free virion elimination rate, *c*, was set to 20 per day, similar to what was found in other RNA virus^61^; the initial concentration of target cells, *T*_0_, was set to 10^8^ cells mL^−1^, a proxy of the liver size in NHPs, the largest solid organ targeted by EBOV^62^; the eclipse phase duration, noted 1/k, ranges between 2 and 15 h^63,64^, and was set to 6 h (*k*= 4 per day). To take into account the different levels of viral challenge, we assumed that the initial viral load was proportional to the size of the inoculum, and we noted *V*_0_ the initial viral load concentration in animals infected by 1000 ffu, such that the initial viral load was equal to *V*_0_, 10×*V*_0_, and 100×*V*_0_ in NHP infected with 10, 100, and 1000 ffu, respectively. Given the limited amount of individuals, no random effects was assumed for the parameters related to CD8 T-cell dynamics, except for the observed concentration of lymphocyte on the day of challenge. A sensitivity analysis was performed to evaluate the impact of the choice of fixed parameters on model predictions (Supplementary Table 4).

### Model validation using rhesus macaques treated with GS-5734

Next we evaluated if the model could also characterize viral dynamics in animals treated with GS-5734. For that purpose we used already published data where 12 rhesus macaques were left untreated or were treated three days after infection with 10 mg kg^−1^ of GS-5734, a potent nucleotide analog polymerase inhibitor^13^. All model parameters were fixed to the values found above, except the constant drug efficacy, *ε*, to account for differences in antiviral efficacy between favipiravir and GS-5734, and the viral infectivity *β*, to account for differences in viral dynamics between cynomolgus and rhesus macaques. The model was fitted to viral load data to estimate *β* and *ε* using individual data provided in ref. ^13^.

### Simulation study

The model was used to evaluate by simulations the impact of various drug efficacy and treatment initiation timings, on viremia and survival. For each scenario 1000 in silico profiles were generated using mlxR package (http://simulx.webpopix.org/mlxr/) using the estimated distribution parameters (Table 1).

### Code availability

The mlxtran code of the final joint model is provided in the Supplementary Software file.

## Electronic supplementary material

Supplementary Information

Supplementary Software

Supplementary Data 1

Description of Additional Supplementary Files

## Data Availability

Virological and pharmacokinetic data used to build the model were already published in refs. ^9,10,13^. The authors declare that all other data supporting the findings of this study are available within the Article and its Supplementary Information files, or are available from the authors upon request.
