# Supplementary material for: Ebola viral dynamics in nonhuman primates provides insights into virus immuno-pathogenesis and antiviral strategies
Source: Nat Commun. 2018 Oct 1;9:4013. doi: 10.1038/s41467-018-06215-z (PMC6167368; doi:10.1038/s41467-018-06215-z)
Supplement: Supplementary file 1 — Supplementary Information [file 41467_2018_6215_MOESM1_ESM.pdf]

Supplementary Information

Ebola viral dynamics in nonhuman primates provides insights into virus immuno-pathogenesis and antiviral strategies

Madelain et al.

Supplementary Tables:

Supplementary Table 1: Infectious titers measured by plaque assay on the day of euthanasia in a subset of infected macaques. The limit of quantitation of the assay was 10 $ffu.mL^{-1}$.

Animal	Dosing ($mg.kg^{-1}$ BID)	Outcome	Titer in liver ($ffu.mL^{-1}$)	Titer in spleen ($ffu.mL^{-1}$)
CCJ046	0	dead	ND	ND
CCC034	0	dead	4,50E+03	1,25E+04
CCC026	150	dead	ND	ND
CCC073	150	dead	ND	2,83E+02
CBK018	150	dead	ND	ND
CA899	150	survivor	ND	ND
CCC003	150	survivor	ND	ND
CAK013	180	dead	ND	ND
CCC027	180	survivor	ND	ND
CBD021	180	survivor	ND	ND
CCD007	180	survivor	ND	ND

Supplementary Table 2: Statistical analysis of plasma cytokines association to viremia and survival time in untreated and treated macaques at D7 post infection.

	Plasma cytokine value at D7 post infection (pg.mL^{-1})						Correlation			
	No treatment (n=10)*		150 mg.kg^{-1} BID (n=5)		180 mg.kg^{-1} BID (n=5)		to viremia at D7		to survival time**	
Cytokine	median	[min-max]	median	[min-max]	median	[min-max]	rho	qvalue	rho	qvalue
CXCL10	13	[0-88]	0	[0-0]	0	[0-0]	0.42	7.40E-01	-0.61	8.80E-02
FGF2	0	[0-142]	0	[0-137]	0	[0-0]	-0.15	8.30E-01	0.01	9.60E-01
G-CSF	46	[26-131]	8	[1-32]	13	[2-21]	0.73	**2.60E-02**	-0.86	**4.40E-04**
GM-CSF	16.5	[11-42]	2	[0-7]	2	[2-2]	0.66	7.50E-02	-0.77	**7.70E-03**
GrzB	0	[0-23]	0	[0-0]	1	[0-3]	-0.06	8.30E-01	-0.07	9.60E-01
IFNα	402	[235.21-678.33]	16.25	[0-376.88]	0	[0-97.71]	0.89	**9.80E-04**	-0.9	**6.20E-04**
IFNγ	290	[106-999]	6	[0-33]	5	[1-16]	0.7	**4.10E-02**	-0.82	**1.80E-03**
IL10	938.5	[59-1558]	17	[5-360]	31	[11-41]	0.75	**1.70E-02**	-0.86	**4.90E-04**
IL15	42.5	[25-66]	5	[2-15]	10	[7-10]	0.65	8.20E-02	-0.78	**5.50E-03**
IL18	110	[36-547]	0	[0-75]	0	[0-14]	0.68	5.70E-02	-0.74	**1.50E-02**
IL1RA	1026	[407-3504]	156	[21-594]	69	[5-112]	0.72	**2.60E-02**	-0.84	**8.90E-04**
IL2	80.5	[59-89]	17	[14-65]	21	[15-35]	0.7	**4.00E-02**	-0.69	**2.40E-02**
IL4	27	[16-57]	1	[0-30]	6	[0-9]	0.54	2.30E-01	-0.69	**2.40E-02**
IL6	158.5	[114-1064]	7	[2-40]	3	[3-3]	0.73	**2.40E-02**	-0.93	**6.60E-06**
IL8	290.5	[74-1246]	225	[19-1024]	662	[404-785]	0.23	8.30E-01	-0.06	9.60E-01
MCP1	3132	[1565-4797]	295	[154-2330]	247	[184-511]	0.73	**2.60E-02**	-0.72	**2.00E-02**
MIP1a	11.5	[7-26]	4	[2-8]	7	[3-7]	0.55	2.30E-01	-0.66	**3.60E-02**
MIP1b	20	[7-33]	3	[0-7]	5	[0-6]	0.75	**1.80E-02**	-0.77	**7.70E-03**
Perforin	31148	[16335-47728]	7917	[2522-48045]	9885	[6585-12641]	0.64	1.10E-01	-0.74	**2.10E-02**
sCD137	1	[0-25]	13	[4-24]	13	[8-14]	-0.3	8.30E-01	0.34	9.60E-01
sCD40L	43.5	[25-103]	14	[5-43]	67	[21-70]	0.11	8.30E-01	-0.31	9.60E-01
TNFα	41.5	[18-85]	6	[0-40]	12	[3-16]	0.56	2.30E-01	-0.7	**2.40E-02**
VEGF	99	[68-222]	34	[21-66]	42	[0-74]	0.41	7.40E-01	-0.71	**2.30E-02**

*n=5 untreated animals for IFNα ; **viremia Spearman correlation to survival time: r = 0.79 , p value = $3.4\ 10^{-5}$

Supplementary Table 3: Design of the non-human primate experiments used to build the PK-VK model.

Infected studies	Infection	Favipiravir dosing	Number	Survival rate at D21	Data
Reaction 0	1000 pfu	none	4	0%	Viral load
	100 pfu	none	4	0%	Viral load
	10 pfu	none	4	0%	Viral load
Reaction 1	1000 pfu	none	3	0%	Viral load
	10 pfu	none	3	0%	Viral load
	1000 pfu	100 $mg.kg^{-1}$ BID	3	0%	Viral load + PK
	10 pfu	100 $mg.kg^{-1}$ BID	3	0%	Viral load + PK
	none	100 $mg.kg^{-1}$ BID	3	100%	PK
Reaction 2	1000 pfu	none	5	0%	Viral load + cytokines
	1000 pfu	150 $mg.kg^{-1}$ BID	5	40%	Viral load + cytokines + PK
	none	150 $mg.kg^{-1}$ BID	4	100%	PK
Reaction 3	1000 pfu	none	5	0%	Viral load + cytokines + cytometry
	1000 pfu	180 $mg.kg^{-1}$ BID	5	60%	Viral load + cytokines + cytometry + PK

Supplementary table 4: Sensitivity analysis on fixed parameters. Estimation was performed with different value for each fixed parameters under the same setting used for the final model, and estimated parameter values were reported. Parameters estimates out of the interval [final model estimate/3 – final model estimate*3] are reported in red.

				c (day^{-1})					k (day^{-1})			T_0 ($cells.mL^{-1}$)				df (day^{-1})			
**Parameter**	**unit**	**Parameter estimates**	**Parameter estimate 95% IC**	**2**	**10**	**40**	**200**	**0.4**	**2**	**8**	**40**	**10^7**	**5.10^7**	**2.10^8**	**10^9**	**0.04**	**0.2**	**0.8**	**4**
Infected elimination rate at baseline δ	day^{-1}	0.224 (9)	[0.18;0.26]	0.24	0.217	0.191	0.233	0.36	0.246	0.189	0.249	0.251	0.202	0.287	0.226	0.195	0.194	0.208	0.209
Viral production p	10^4 $Virion.cell^{-1}.day^{-1}$	4.15 (71)	[-1.63;9.93]	0.716	2.35	6.67	32.9	3.46	3.26	3.72	2.91	2.15	3.86	8.33	7.82	7.79	9.90	3.96	0.494
Virion elimination rate c	day^{-1}	20	_	2	10	40	200	20	20	20	20	20	20	20	20	20	20	20	20
Initial pool of target cells T_0	$cells.mL^{-1}$	10^8	_	10^8	10^8	10^8	10^8	10^8	10^8	10^8	10^8	10^7	$10^{7.7}$	$10^{8.3}$	10^9	10^8	10^8	10^8	10^8
Inoculum V_0	10^{-5} $virion.mL^{-1}$	7.9 (11)	[6.2;9.6]	0.479	2.04	4.90	10.7	0.398	7.08	1.15	2.51	2.82	8.91	13.5	3.98	1.17	1.70	2.69	7.94
Infectivity constant β	10^{-11} $mL.virion^{-1}.day^{-1}$	7.9 (73)	[-3.4;19.2]	15.2	9.42	9.78	9.69	84.5	16.1	7.50	6.08	175	16.9	1.87	0.427	4.93	3.84	8.96	63.8
Eclipse phase constant k	day^{-1}	4	_	4	4	4	4	0.4	2	8	40	4	4	4	4	4	4	4	4

Favipiravir maximal effect E_{max}	_	1	_	1	1	1	1	1	1	1	1	1	1	1	1	1	1	1	1
In vivo favipiravir EC_{50}	µg.mL^{-1}	191 (20)	[116;266]	182	159	176	164	171	184	194	213	156	189	198	272	238	183	210	281
IFNα production rate q	10^{-3} pg.cell^{-1}.day^{-1}	7.40 (69)	[-2.61;17.4]	12.8	10.7	7.50	5.33	9.65	6.47	6.32	6.26	5.55	10.3	11.3	11.8	8.45	10.5	8.60	7.14
IFNα elimination rate d_F	day^{-1}	0.4	_	0.4	0.4	0.4	0.4	0.4	0.4	0.4	0.4	0.4	0.4	0.4	0.4	0.04	0.2	0.8	4
IFNα concentration providing 50% of max effect on refractory conversion θ_T	pg.mL^{-1}	1.73 (35)	[0.54;2.92]	0.765	0.58	0.994	0.697	0.116	0.958	0.425	1.52	0.423	0.978	2.46	0.687	0.282	0.526	0.593	0.364
Target to refractory cell conversion constant ϕ	mL.pg^{-1}.day^{-1}	2.67 (17)	[1.78;3.56]	2.65	2.03	2.33	2.25	2.51	2.64	1.89	1.76	1.76	1.89	2.95	2.24	2.19	2.6	2.08	1.23
CD8 T cell perforin+ baseline value C_0	10^4 cell.mL^{-1}	3.69 (30)	[1.52;5.86]	4.62	3.91	4.49	5.02	4.46	4.53	4.61	3.92	4.66	3.38	3.47	3.69	4.15	4.87	3.77	4.19
Initial proportion of specific CD8 T cell perforin+ P_0	_	0.001	_	0.001	0.001	0.001	0.001	0.001	0.001	0.001	0.001	0.001	0.001	0.001	0.001	0.001	0.001	0.001	0.001
CD8 T cell perforin+ elimination rate δ_E	day^{-1}	0.001	_	0.001	0.001	0.001	0.001	0.001	0.001	0.001	0.001	0.001	0.001	0.001	0.001	0.001	0.001	0.001	0.001
CD8 T cell perforin+ elimination rate mediated by viremia ζ	mL.virion^{-1}.day^{-1}	0.455 (41)	[0.09;0.82]	0.362	0.35	0.295	0.3	0.321	0.287	0.331	0.275	0.422	0.463	0.388	0.332	0.313	0.367	0.28	0.297
Specific CD8 T cell perforin+ growth constant ρ	day^{-1}	0.338 (11)	[0.27;0.41]	0.372	0.408	0.344	0.305	0.332	0.389	0.369	0.384	0.417	0.437	0.437	0.431	0.404	0.353	0.323	0.353
Non specific CD8 T cell perforin+ growth constant σ	10^3 cell.mL^{-1}.day^{-1}	3.05 (87)	[-2.15;8.25]	2.22	1.49	1.76	1.40	1.91	1.33	1.45	1.89	2.22	3.80	2.30	1.94	0.945	1.21	1.53	1.33
IFNα concentration providing 50% of max effect on CD8 T cell perforin+ depletion θ_E	10^{-4} pg.mL^{-1}	6.5 (233)	[-23.2;36.2]	3.15	5.94	1.62	2.30	6.64	5.32	2.33	2.15	7.30	0.861	4.31	2.34	4.12	3.12	3.19	3.50
CD8 T cell perforin+ mediated infected cell elimination rate κ	10^{-5} day^{-1}.cell^{-1}.mL^{-1}	2.08 (31)	[0.82;3.34]	5.10	3.34	4.68	5.71	5.76	3.02	3.98	3.54	2.16	6.29	3.88	3.02	3.02	3.55	5.46	3.80
IL6 production rate q_L	10^{-3} pg.cell^{-1}.day^{-1}	9.7 (65)	[-2.66;22.1]	14	12	9	8	11	7	10	7	6	9	17	17	10	17	17	9
IL6 elimination rate d_L	day^{-1}	0.4	_	0.4	0.4	0.4	0.4	0.4	0.4	0.4	0.4	0.4	0.4	0.4	0.4	0.04	0.2	0.8	4
TNFα production rate q_N	10^{-3} pg.cell^{-1}.day^{-1}	4.6 (69)	[-1.62;10.8]	6.0	5.5	3.9	3.4	5.1	3.4	4.6	3.5	3.4	4.0	7.6	9.0	4.2	7.2	7.2	4.1
TNFα elimination rate d_N	day^{-1}	0.4	_	0.4	0.4	0.4	0.4	0.4	0.4	0.4	0.4	0.4	0.4	0.4	0.4	0.04	0.2	0.8	4
-2loglikelihood OF	_	1334	_	1341	1334	1334	1348	1356	1340	1337	1340	1337	1343	1343	1339	1326	1324	1374	1391
Bayesian information criterion BIC	_	1440	_	1447	1440	1446	1454	1462	1446	1443	1446	1443	1449	1449	1445	1432	1430	1480	1497

Supplementary table 5: Infection and center effect assessment on favipiravir pharmacokinetics model parameters.

cov	parameter	ΔBIC	cov	parameter	ΔBIC	cov	parameter	ΔBIC
**BSL4**	$\boldsymbol{R_{in}}$	**-24.3**	infection	R_{in}	-8.9	infection+ BSL4	R_{in}	-12.8
**BSL4**	k	3.0	infection	k	2.3	infection+ BSL4	k	9.6
**BSL4**	α_{deg}	1.6	infection	α_{deg}	6.0	infection+ BSL4	α_{deg}	6.2
**BSL4**	λ	3.7	infection	λ	7.1	infection+ BSL4	λ	9.4
**BSL4**	Vd	4.9	infection	Vd	4.4	infection+ BSL4	Vd	6.6
**BSL4**	k_{enz}	-2.3	infection	k_{enz}	2.8	infection+ BSL4	k_{enz}	2.2

Supplementary table 6: Parameter estimates of the target cell limited model

			Fixed effect		Sd of the random effect	
parameter	name	unit	estimate	r.s.e.(%)	estimate	r.s.e.(%)
Infected elimination rate at baseline	δ	day^{-1}	0.667	9	0.156	48
Viral production	p	virion.cell^{-1}.day^{-1}	27	45	2.59	12
Virion elimination rate	c	day^{-1}	20	-	0	-
Initial pool of target cells	T_0	cells.mL^{-1}	10^{8}	-	0	-
Inoculum	V_0	virion.mL^{-1}	$10^{-3.59}$	10	1	-
Infectivity constant	β	mL.virion^{-1}.day^{-1}	$1.09\ 10^{-7}$	47	0.121	87
Eclipse phase constant	k	day^{-1}	4	-	0	-
Favipiravir maximal effect	E_{max}		1	-	0	-
In vivo favipiravir EC_{50}	EC_{50}	µg.mL^{-1}	241	20	0	-
Viremia additive residual error	a	Log10 virion.mL^{-1}	0.88	7	-	-

Supplementary table 7: Candidate models incorporating innate immune response compartment. Compared to the target cell limited model, these models added the following parameters: κ the infected cell elimination rate driven by innate response, ϕ the maximal rate of at which susceptible cells are refractory to infection, θ_T the cytokine concentration providing half of the maximal effect, q is the apparent cytokine production per infected cell, and d_F the apparent cytokine elimination rate. The initial conditions are $T_{(t=0)} = T_0;\ I_{1(t=0)} = 0;\ I_{2(t=0)} = 0;\ V_{(t=0)} = V_0;\ F_{(t=0)} = 0$ for all the models.

	**Refractory model**	**Target production model**	**Viral prod inhibition model**	**Cytotoxic model**
$\frac{dT}{dt} =$	$-\beta TV - \frac{\phi TF}{F+\theta_T}$	$-\beta TV + \frac{\phi TF}{F+\theta_T}$	$-\beta TV$	$-\beta TV$
$\frac{dI_1}{dt} =$	$\beta TV - kI_1$	$\beta TV - kI_1$	$\beta TV - kI_1$	$\beta TV - kI_1$
$\frac{dI_2}{dt} =$	$kI_1 - \delta I_2$	$kI_1 - \delta I_2$	$kI_1 - \delta I_2$	$kI_1 - \delta I_2 - \frac{\delta_2 I_2 F}{F+\theta_T}$
$\frac{dV}{dt} =$	$p(1-\varepsilon)I_2 - cV$	$p(1-\varepsilon)I_2 - cV$	$p(1-\varepsilon)(1-\frac{F}{F+\theta_T})I_2 - cV$	$p(1-\varepsilon)I_2 - cV$
$\frac{dF}{dt} =$	$qI_2 - d_F F$	$qI_2 - d_F F$	$qI_2 - d_F F$	$qI_2 - d_F F$

Supplementary table 8: Selection procedure of the mechanistic model including innate response compartment. OF objective function.

Cytokine	Candidate model	viremia OF	cytokine OF
None	target cell limited	645.7	_
**IL6**	**refractory model (i)**	**618.0**	**202**
IL6	target cells production model (ii)	644.8	187.4
IL6	inhibition of viral production model (iii)	645.7	195.8
**IFNα**	**refractory cells model (i)**	**622.4**	**218.3**
IFNα	target cells production model (ii)	641.8	225.9
IFNα	inhibition of viral production model (iii)	692.8	225.5
**TNFα**	**refractory cells model (i)**	**621.8**	**264.6**
TNFα	target cells production model (ii)	633.1	223.8
TNFα	inhibition of viral production model (iii)	633.3	215.1
IFNγ	Cytotoxic model (iv)	634.5	226.2
IL2	Cytotoxic model (iv)	636.3	-49.7
perforin	Cytotoxic model (iv)	634.6	-2.6
IL15	Cytotoxic model (iv)	634.6	81.6
IL18	Cytotoxic model (iv)	634.8	255.3

Supplementary table 9: Parameter estimates of the model assuming innate response compartment converting target cells into refractory cells, fitted to the plasma EBOV viral load, IFNα, IL6 and TNFα data in infected macaques.

			Fixed effect		Sd of the random effect	
parameter	name	unit	estimate	r.s.e.(%)	estimate	r.s.e.(%)
Infected elimination rate at baseline	δ	day^{-1}	0.708	7	0.21	19
Viral production	p	$virions.cell^{-1}.day^{-1}$	$5.6\ 10^{5}$	92	0.978	90
Virion elimination rate	c	day^{-1}	20	-	0	-
Initial pool of target cells	T_0	$cells.mL^{-1}$	10^{8}	-	0	-
Inoculum	V_0	$virion.mL^{-1}$	$10^{-4.3}$	9	1	-
Infectivity constant	β	$mL.virion^{-1}.day^{-1}$	$6.39\ 10^{-12}$	13	0.093	96
Eclipse phase constant	k	day^{-1}	4	-	0	-
Favipiravir maximal effect	E_{max}		1	-	0	-
In vivo favipiravir EC_{50}	EC_{50}	$\mu g.mL^{-1}$	247	20	0	-
IFNα production rate	q	$pg.cell^{-1}.day^{-1}$	0.148	101	0	-
IFNα elimination rate	d_F	day^{-1}	0.4	-	0	-
IFNα concentration providing 50% of max effect on refractory conversion	θ_T	$pg.mL^{-1}$	0.446	52	0.973	38
Target to refractory cell conversion constant	ϕ	$mL.pg^{-1}.day^{-1}$	1.31	12	0.080	270
Viremia additive residual error	a_V	Log10 $virion.mL^{-1}$	0.86	6		
IFNα additive residual error	a_F	Log10 $pg.mL^{-1}$	1.05	8		

Supplementary table 10: Selection procedure of the mechanistic model including innate response and adaptive cytotoxic response compartments. OF objective function.

CD8 T cell population	Candidate model	Viremia OF	Cytokine +CD8 OF
_	Target cell limited	645.7	_
_	Refractory	618	202
**Expressing perforin**	**Refractory cytotoxic**	**605.5**	**275.2**
Expressing granzymeB	Refractory cytoxic	611.6	268.7
Expressing NKp80	Refractory cytoxic	608.4	247.6

Supplementary table 11: Final viral kinetic model parameter estimates

			Fixed effect		Sd of the random effect	
parameter	name	unit	estimate	r.s.e.(%)	estimate	r.s.e.(%)
Infected elimination rate at baseline	δ	day^{-1}	0.224	9	0.169	40
Viral production	p	virion.cell^{-1}.day^{-1}	4.15 10^{4}	71	1.76	18
Virion elimination rate	c	day^{-1}	20	-	0	-
Initial pool of target cells	T_0	cells.mL^{-1}	10^{8}	-	0	-
Inoculum	V_0	virion.mL^{-1}	$10^{-4.1}$	11	1.5	17
Infectivity constant	β	mL.virion^{-1}.day^{-1}	7.9 10^{-11}	73	0	-
Eclipse phase constant	k	day^{-1}	4	-	0	-
Favipiravir maximal effect	E_{max}		1	-	0	-
In vivo favipiravir EC50	EC_{50}	µg.mL^{-1}	191	20	0	-
IFNα production rate	q	pg.cell^{-1}.day^{-1}	0.0074	69	0	-
IFNα elimination rate	d_F	day^{-1}	0.4	-	0	-
IFNα concentration providing 50% of max effect on refractory conversion	θ_T	pg.mL^{-1}	1.73	35	1.05	16
Target to refractory cell conversion constant	ϕ	mL.pg^{-1}.day^{-1}	2.67	17	0	-
CD8 T cell perforin+ baseline value	C_0	cell.mL^{-1}	36900	30	0.775	23

Initial proportion of specific CD8 T cell perforin+	P_0		0.001	-	0	-
CD8 T cell perforin+ elimination rate	δ_E	day^{-1}	0.001	-	0	-
CD8 T cell perforin+ elimination rate mediated by IFNα	ζ	$mL.virion^{-1}.day^{-1}$	0.455	41	0	-
Specific CD8 T cell perforin+ growth constant	ρ	day^{-1}	0.338	11	0	-
Non specific CD8 T cell perforin+ growth constant	σ	$cell.mL^{-1}.day^{-1}$	3050	87	0	-
IFNα concentration providing 50% of max effect on lymphocyte depletion	θ_E	$pg.mL^{-1}$	$6.5\ 10^{-4}$	233	0	-
CD8 T cell perforin+ mediated infected cell elimination rate	κ	$mL\ .day^{-1}.cell^{-1}$	$2.08\ 10^{-5}$	31	0	-
IL6 production rate	q_L	$pg.cell^{-1}.day^{-1}$	0.0097	65	0.76	37
IL6 elimination rate	d_L	day^{-1}	0.4	-	0	-
TNFα production rate	q_N	$pg.cell^{-1}.day^{-1}$	0.0046	69	1.03	30
TNFα elimination rate	d_N	day^{-1}	0.4	-	0	-
Viremia additive residual error	a_V	Log10 $virion.mL^{-1}$	0.82	7	-	-
IFNα additive residual error	a_F	Log10 $pg.mL^{-1}$	1.08	6	-	-
IL6 additive residual error	a_L	Log10 $pg.mL^{-1}$	0.47	7	-	-
TNFα additive residual error	a_N	Log10 $pg.mL^{-1}$	0.36	11	-	-
CD8 T cell perforin+ residual error	a_E	Log10 $cell.mm^{-3}$	0.68	14	-	-

Supplementary table 12: Selection procedure of the joint model. OF objective function, BIC Bayesian information criterion.

Link to the hazard function	Model compartment driving the hazard of death	OF	BIC
effect compartment	viremia	1580.3	1591.7
**effect compartment**	**IL6**	**1495.0**	**1506.3**
**effect compartment**	**IFNα**	**1495.7**	**1507.0**
effect compartment	TNFα	1510.8	1522.1

Supplementary table 13: Parameter estimates of the joint model assuming innate response compartment converting target cells into refractory cells and adaptive response increasing infected cell elimination rate, fitted to the plasma EBOV viral load, IFNα, IL6, TNFα and CD8 T cell expressing perforin data in infected macaques, and assuming hazard ratio depending on effect compartment of IFNα.

			Fixed effect		Sd of the random effect	
parameter	name	unit	estimate	r.s.e.(%)	estimate	r.s.e.(%)
Infected elimination rate at baseline	δ	day^{-1}	0.224	-	0.169	-
Viral production	p	$virion.cell^{-1}.day^{-1}$	$4.15\ 10^{4}$	-	1.76	-
Virion elimination rate	c	day^{-1}	20	-	0	-
Initial pool of target cells	T_0	$cells.mL^{-1}$	10^{8}	-	0	-
Inoculum	V_0	$virion.mL^{-1}$	$10^{-4.1}$	-	1.5	-
Infectivity constant	β	$mL.virion^{-1}.day^{-1}$	$7.9\ 10^{-11}$	-	0	-
Eclipse phase constant	k	day^{-1}	4	-	0	-
Favipiravir maximal effect	E_{max}		1	-	0	-
In vivo favipiravir EC50	EC_{50}	$\mu g.mL^{-1}$	191	-	0	-
IFNα production rate	q	$pg.cell^{-1}.day^{-1}$	0.0074	-	0	-
IFNα elimination rate	d_F	day^{-1}	0.4	-	0	-
IFNα concentration providing 50% of max effect on refractory conversion	θ_T	$pg.mL^{-1}$	1.73	-	1.05	-
Target to refractory cell conversion constant	ϕ	$mL.pg^{-1}.day^{-1}$	2.67	-	0	-
CD8 T cell perforin+ baseline value	C_0	$cell.mL^{-1}$	36900	-	0.775	-
Initial proportion of specific CD8 T cell perforin+	P_0		0.001	-	0	-
CD8 T cell perforin+ elimination rate	δ_E	day^{-1}	0.001	-	0	-
CD8 T cell perforin+ elimination rate mediated by IFNα	ζ	$mL.virion^{-1}.day^{-1}$	0.455	-	0	-
Specific CD8 T cell perforin+ growth constant	ρ	day^{-1}	0.338	-	0	-
Nonspecific CD8 T cell perforin+ growth constant	σ	$cell.mL^{-1}.day^{-1}$	3050	-	0	-
IFNα concentration providing 50% of max effect on CD8 T cell perforin+ depletion	θ_E	$pg.mL^{-1}$	$6.5\ 10^{-4}$	-	0	-
CD8 T cell perforin+ mediated inf cell elimination rate	κ	$mL\ .day^{-1}.cell^{-1}$	$2.08\ 10^{-5}$	-	0	-
IL6 production rate	q_L	$pg.cell^{-1}.day^{-1}$	0.0097	-	0.76	-
IL6 elimination rate	d_L	day^{-1}	0.4	-	0	-
TNFα production rate	q_N	$pg.cell^{-1}.day^{-1}$	0.0046	-	1.03	-
TNFα elimination rate	d_N	day^{-1}	0.4	-	0	-
Maximal hazard of death	λ_m	day^{-1}	1.12	2	0	-

IFNα effect compartment concentration inducing 50% of the maximal hazard	F_{50}	pg.mL^{-1}	103	12	0	-
Transfer constant	k_s	day^{-1}	0.319	3	0	-
Hill coefficient	γ		2	-	-	-
Viremia additive residual error	a_V	Log10 virion.mL^{-1}	0.82	-	-	-
IFNα additive residual error	a_F	Log10 pg.mL^{-1}	1.08	-	-	-
IL6 additive residual error	a_L	Log10 pg.mL^{-1}	0.47	-	-	-
TNFα additive residual error	a_N	Log10 pg.mL^{-1}	0.36	-	-	-
CD8 T cell perforin+ residual error	a_E	Log10 cell.mm^{-3}	0.68	-	-	-

Supplementary Figures:

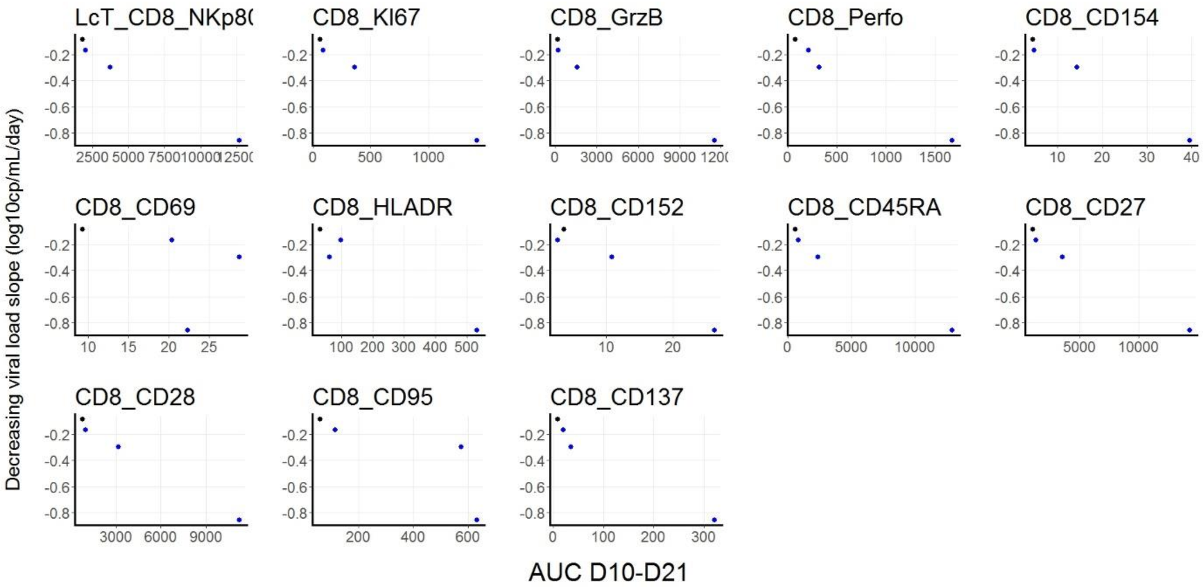

Supplementary Figure 1: Slope of viral decline from D10 to D21 according to the area under the curve from D10 to D21 post challenge of various CD8 T cells populations in the 4 macaques receiving favipiravir 180 mg.kg^{-1} BID and surviving more than 12 days.

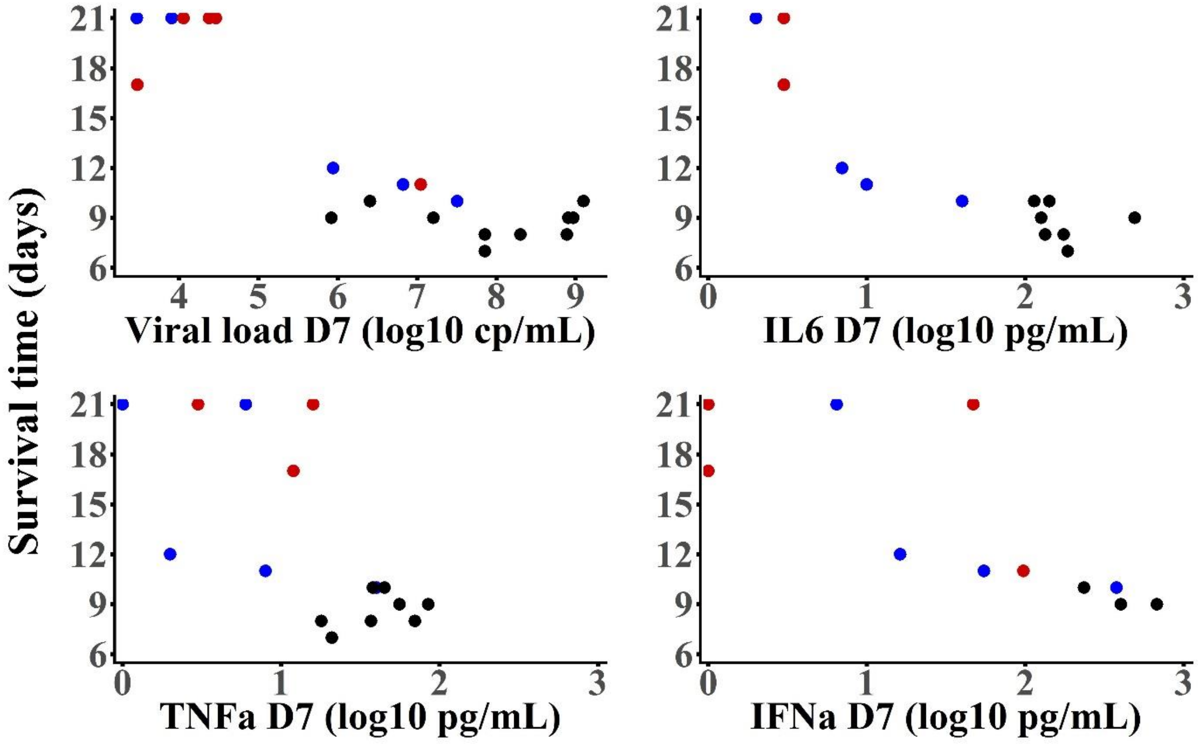

Supplementary Figure 2: Survival times according to viral load and plasma cytokine levels at day 7. Dosing regimen groups were untreated (black), 100 mg.kg^{-1} BID (green), 150 mg.kg^{-1} BID (blue) and 180 mg.kg^{-1} BID (red)(viremia, r = -0.79; IL6, r = -0.93; IFNα, r = -0.9; TNFα, r= -0.7).

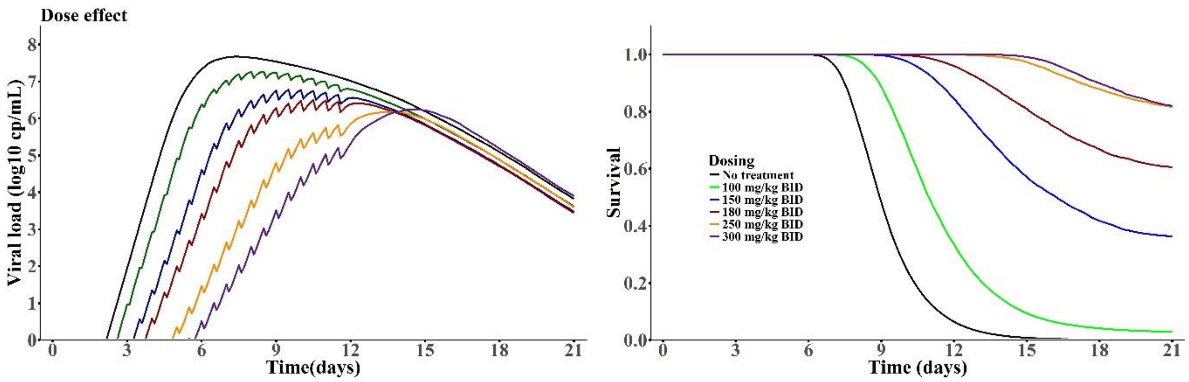

Supplementary Figure 3: Viral load and survival probability predictions in macaques treated with various dosing of favipiravir, evaluated (100, 150 and 180 mg.kg^{-1} BID) in NHP experiments or predicted (250 and 300 mg.kg^{-1} BID).

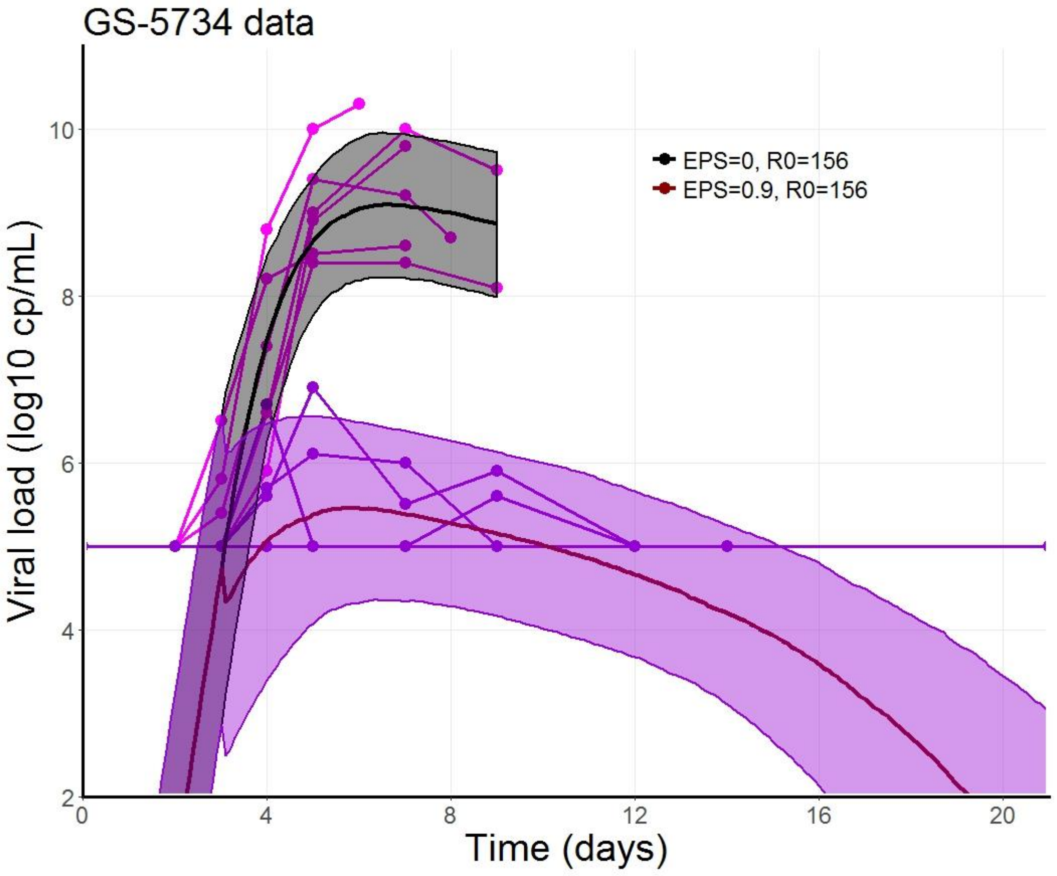

Supplementary Figure 4: Observed viral load data in rhesus macaques infected by Ebola virus. Macaques were left untreated (magenta circles) or treated at day 3 post infection with GS-5734 10 mg.kg^{-1} (purple circles)[1] and model prediction of the model assuming β=8.9 10^{-11} mL.virion^{-1}.day^{-1} and no treatment (plain black line and area) or treatment with ε=88% (plain purple line and area). Results show the median, 10th and 90th percentiles of 1000 simulations.

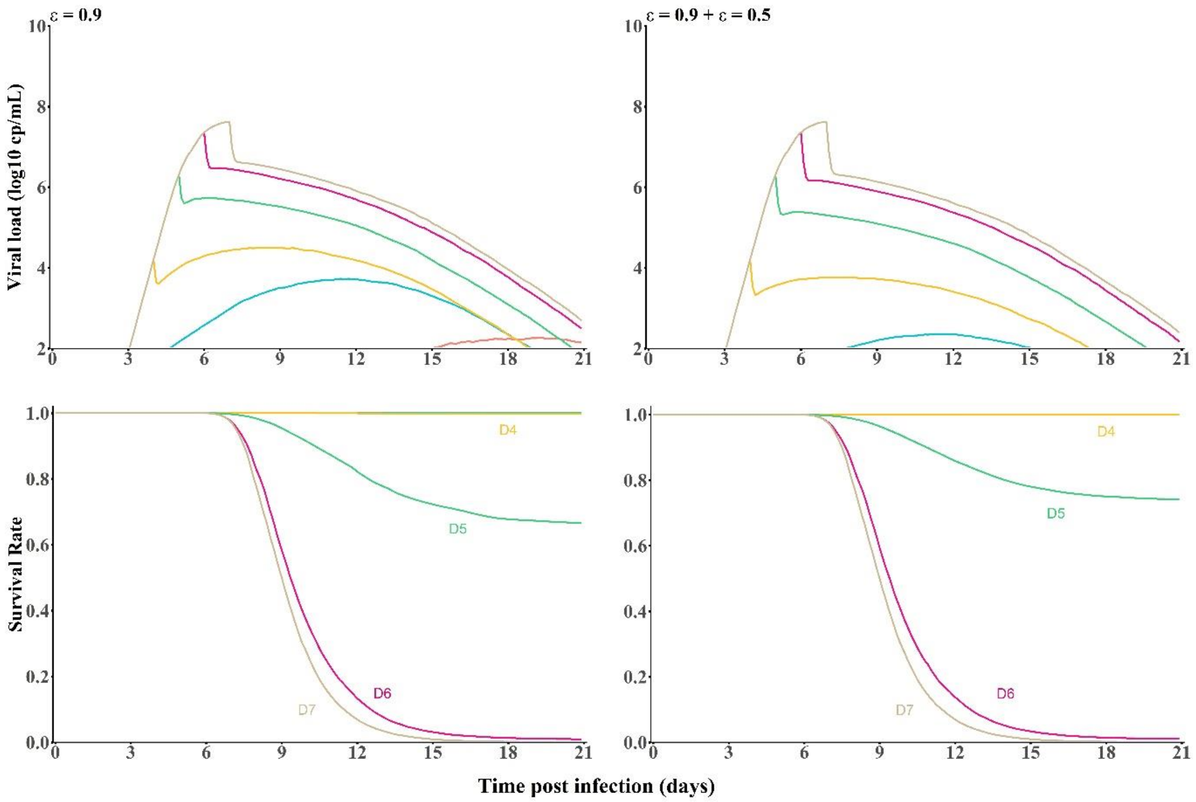

Supplementary Figure 5: Viral load and survival probability predictions in macaques treated with highly effective mono or bitherapy. Macaques were treated with one highly active drug (ε=90%, eg GS-5734, left panels) or combination of one highly active drug and one moderate active drug (ε=50%, eg favipiravir), assuming a Bliss independence model (overall potency = 0.95, right panels) , according to the time of treatment initiation (dark blue D0, salmon D1, light blue D3, yellow D4, green D5, magenta D6 and brown D7 post infection). Results show the median of 1000 simulations.

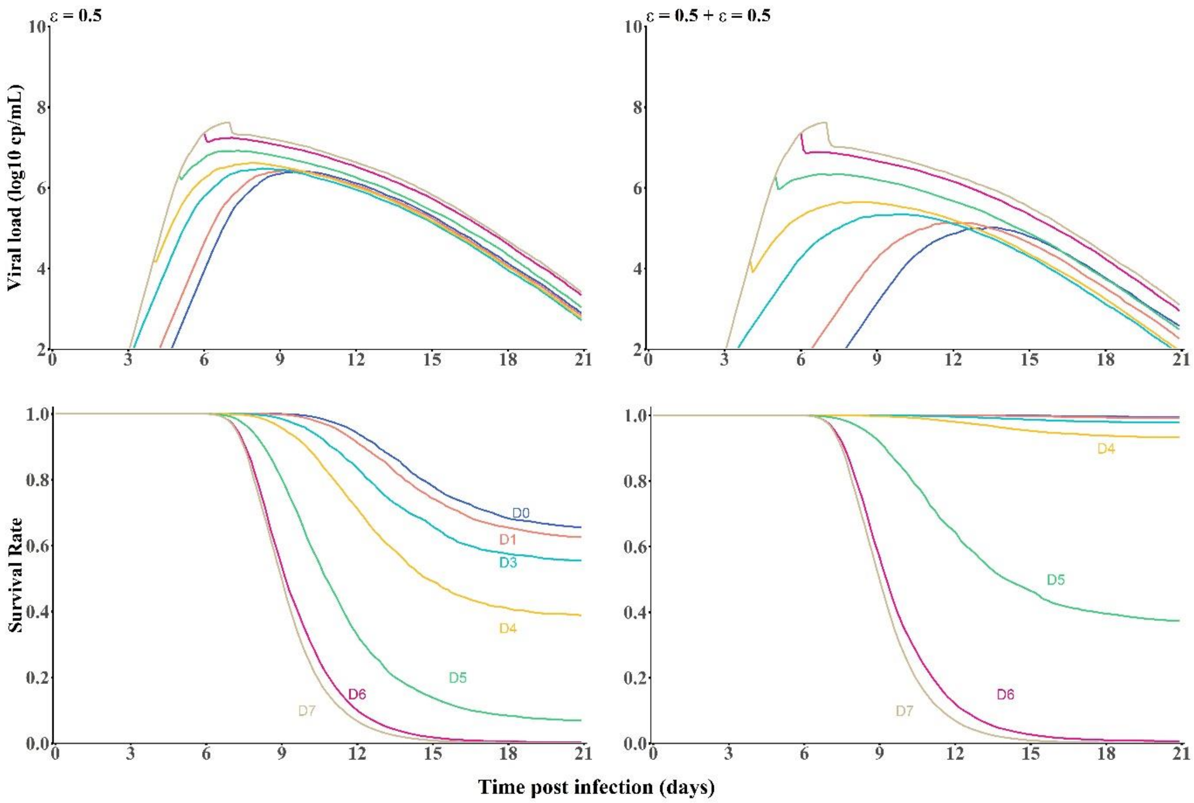

Supplementary Figure 6: Viral load and survival probability predictions in macaques treated with moderate effective mono or bitherapy.. Macaques were treated with one moderately active drug (ε=50%, eg favipiravir, left panels) or co-administration of two moderately active drugs with similar effectiveness, assuming a Bliss independence model (overall potency = 0.75, right panels), according to the time of treatment initiation (dark blue D0, salmon D1, light blue D3, yellow D4, green D5, magenta D6 and brown D7 post infection). Results show the median of 1000 simulations.

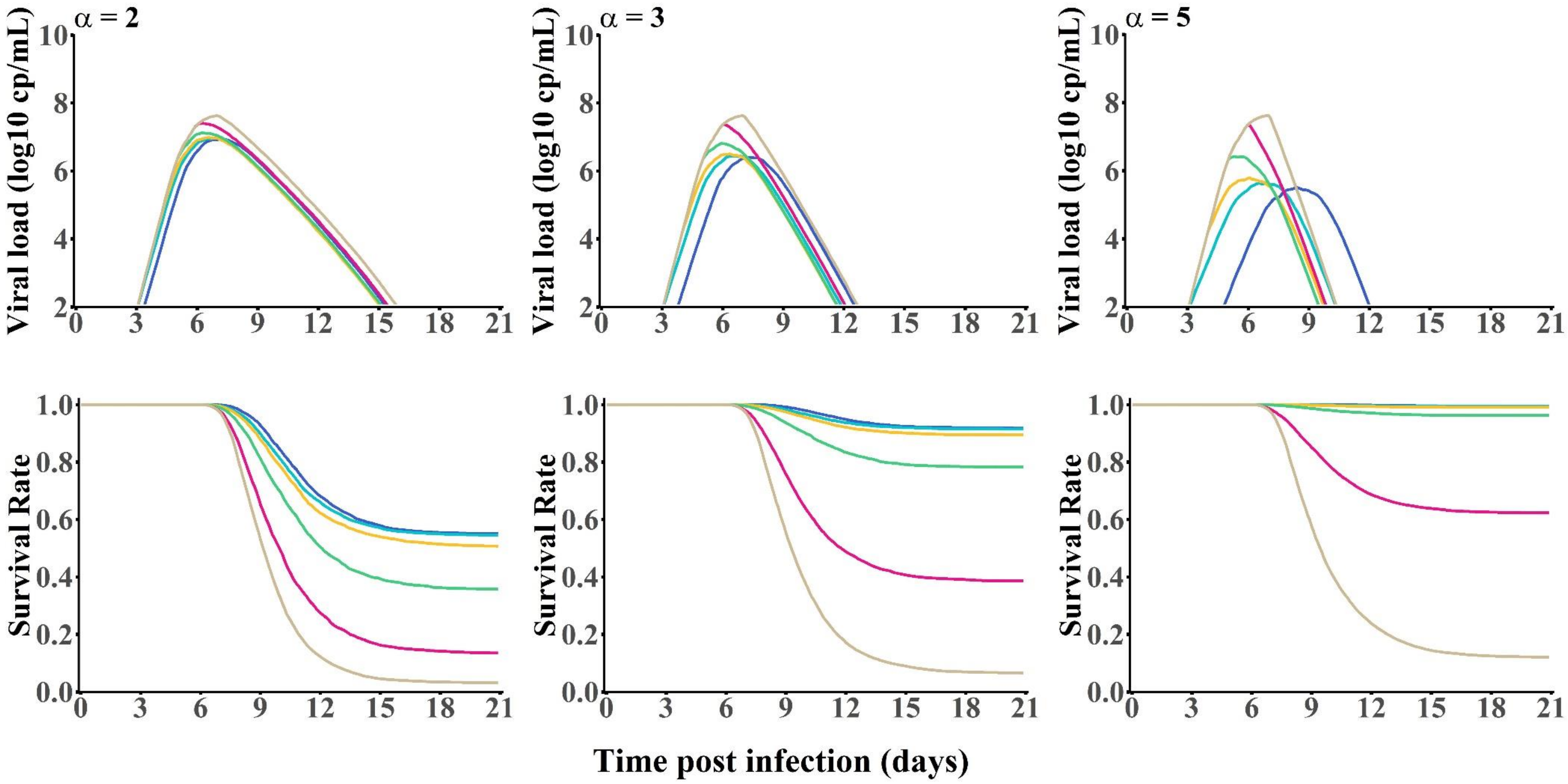

Supplementary Figure 7: Viral load and survival probability predictions in macaques treated with drug of various potency assumed to increase the elimination rate of infected cells, i.e. monoclonal antibodies. Simulation was performed assuming various levels of increase of infected cell elimination rate (α=x2, left panel; α=x3, middle panels and α=x5, right panel), according to the time of treatment initiation (dark blue D0, salmon D1, light blue D3, yellow D4, green D5, magenta D6 and brown D7 post infection). Results show the median of 1000 simulations.

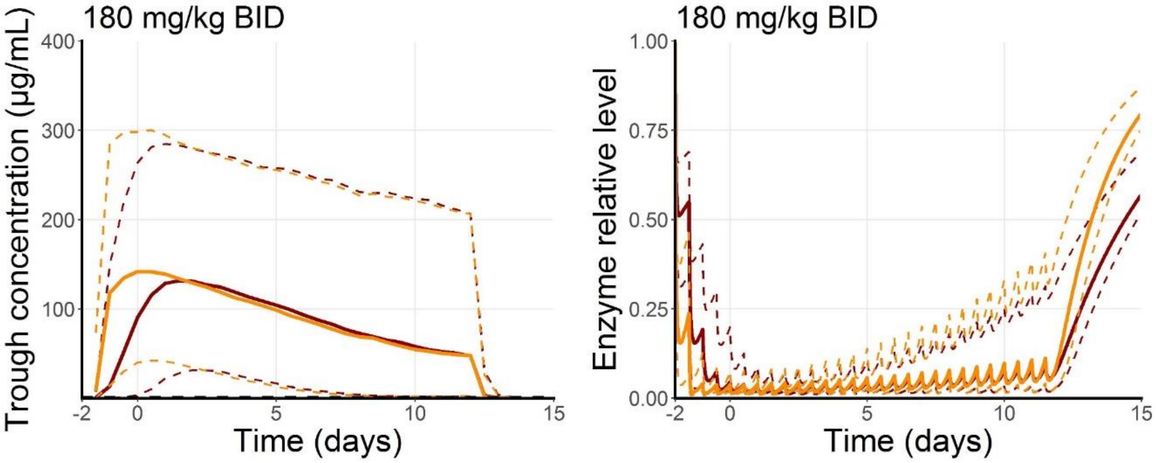

Supplementary Figure 8: Effect of BSL4 center on favipiravir pharmacokinetics. Median [10e-90e percentile] favipiravir trough concentration (left panel) and relative aldehyde oxidase activity (right panel) predicted by the model, in BSL4 macaques (red) and non BLS4 macaques (orange).

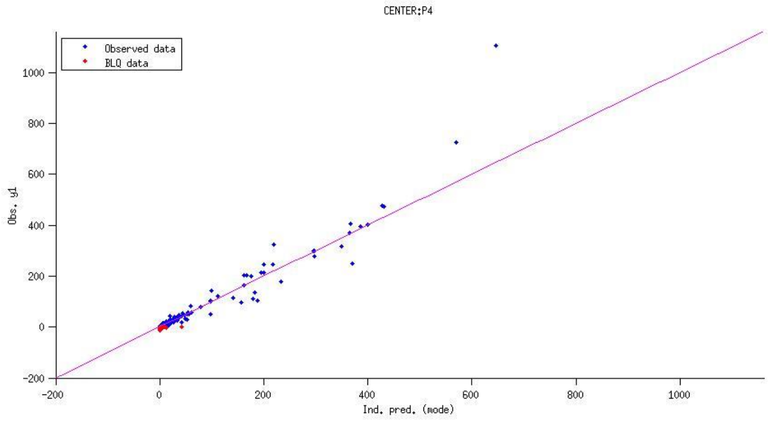

Supplementary Figure 9: observations vs individual predictions in BSL4 macaques (blue observed data, red data below the limit of quantitation).

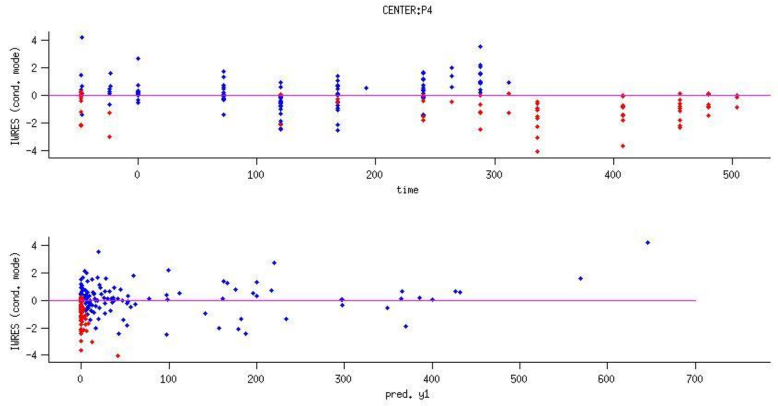

Supplementary Figure 10: individual weighted residuals vs time and vs prediction in BSL4 macaques (blue observed data, red data below the limit of quantitation).

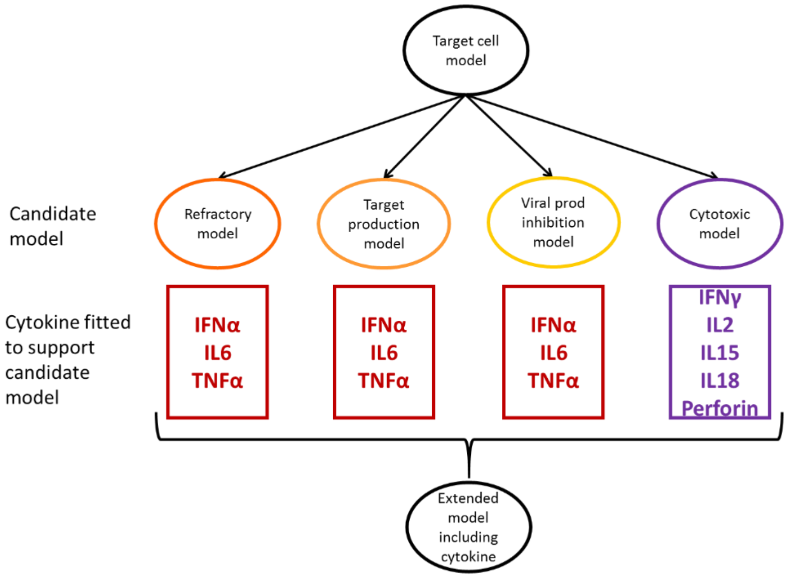

Supplementary Figure 11: Mechanistic model including innate response tested for each cytokine

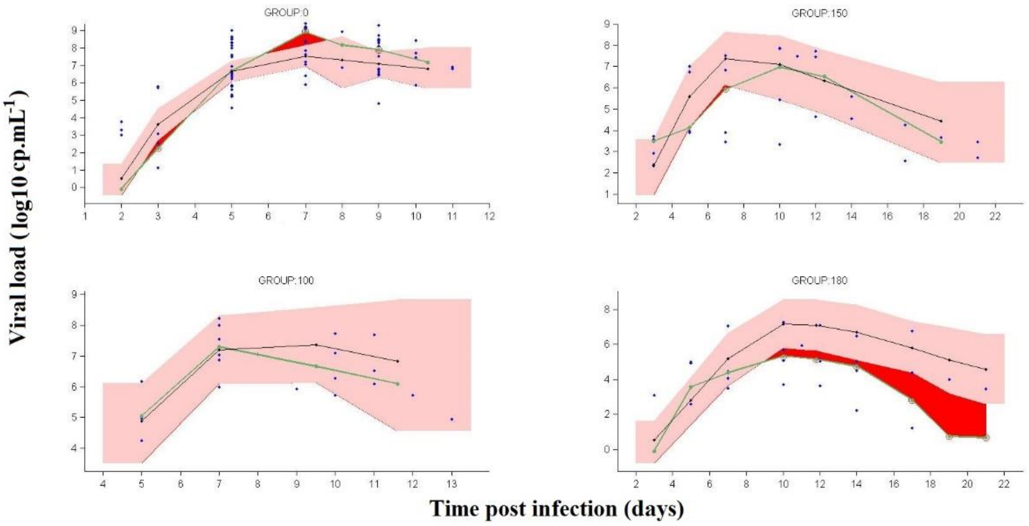

Supplementary Figure 12: Visual predictive check (VPC) per dose of the target cell limited model. The green lines are the observed median, the blue lines are the predicted median, the circles are the individual observations, and the pink area is the 95% confidence interval of the median. The red area indicates where the prediction falls outside this interval and suggests a discrepancy between the observations and the model predictions.

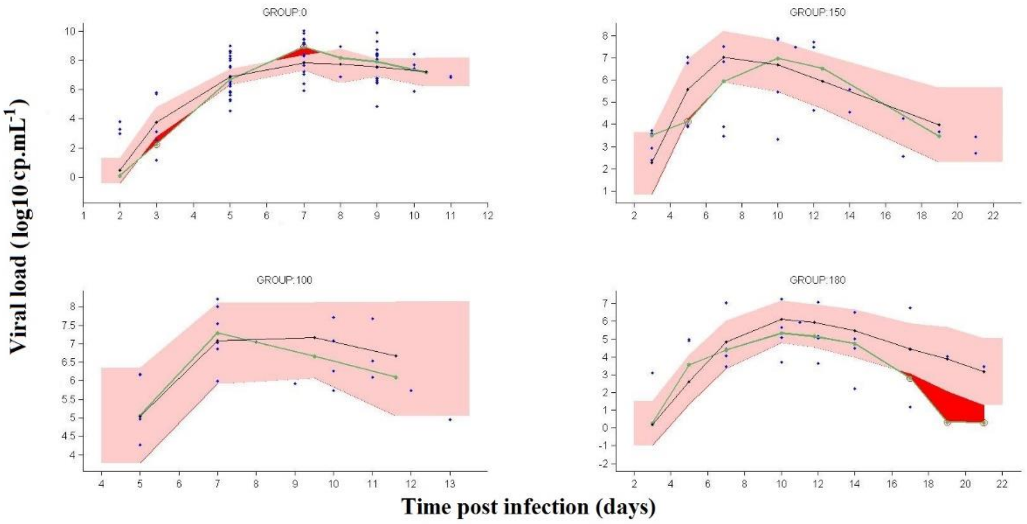

Supplementary Figure 13: VPC per dose of the refractory model fitted with IFNα data. The green lines are the observed median, the blue lines are the predicted median, the circles are the individual observations, and the pink area is the 95% confidence interval of the median. The red area indicates where the prediction falls outside this interval and suggests a discrepancy between the observations and the model predictions.

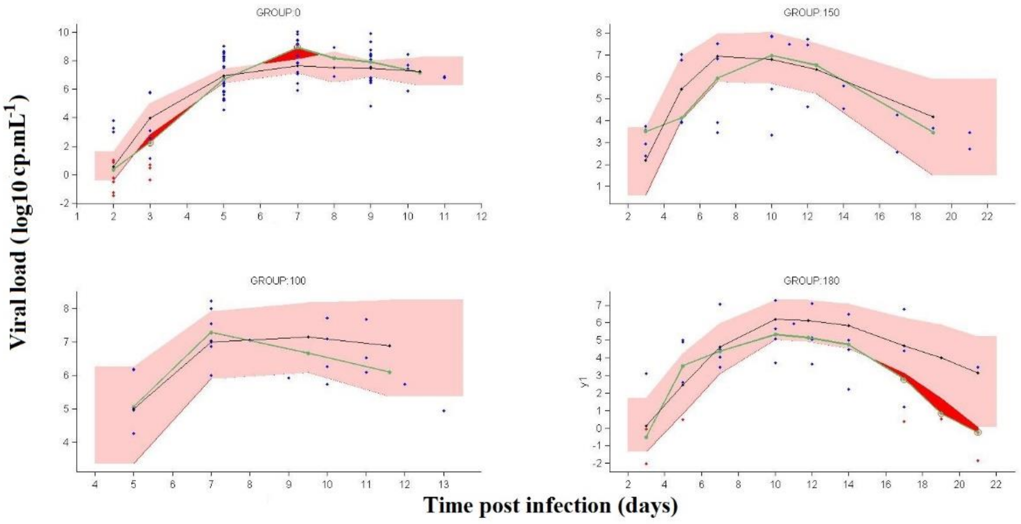

Supplementary Figure 14: VPC per dose of the adaptive response model fitted with the CD8 T cell expressing perforin data. The green lines are the observed median, the blue lines are the predicted median, the circles are the individual observations, and the pink area is the 95% confidence interval of the median. The red area indicates where the prediction falls outside this interval and suggests a discrepancy between the observations and the model predictions.

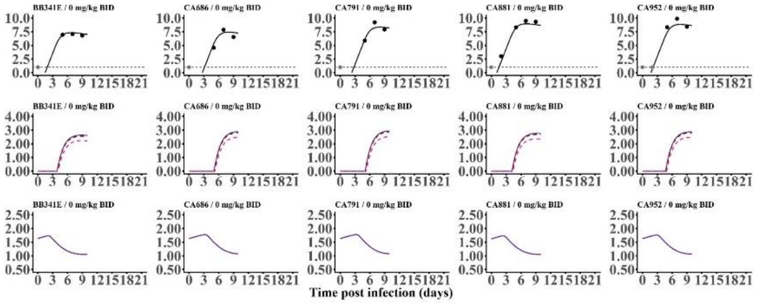
BB341E / 0 mg/kg BID
CA686 / 0 mg/kg BID
CA791 / 0 mg/kg BID
CA881 / 0 mg/kg BID
CA952 / 0 mg/kg BID
Time post infection (days)
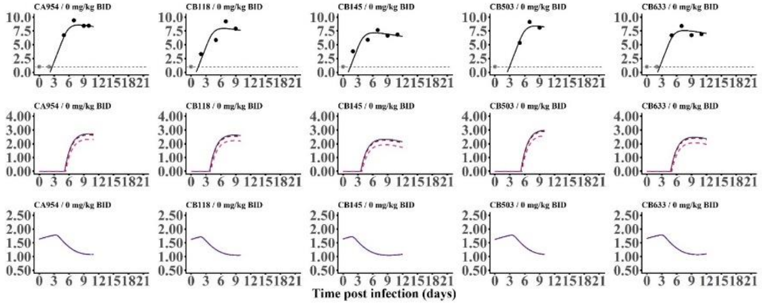
CA954 / 0 mg/kg BID
CB118 / 0 mg/kg BID
CB145 / 0 mg/kg BID
CB503 / 0 mg/kg BID
CB633 / 0 mg/kg BID
Time post infection (days)
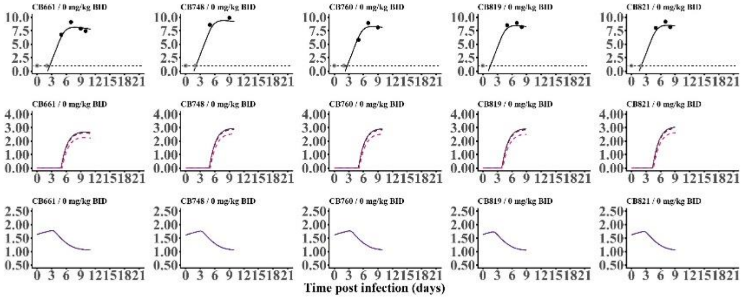
CB661 / 0 mg/kg BID
CB748 / 0 mg/kg BID
CB760 / 0 mg/kg BID
CB819 / 0 mg/kg BID
CB821 / 0 mg/kg BID
Time post infection (days)
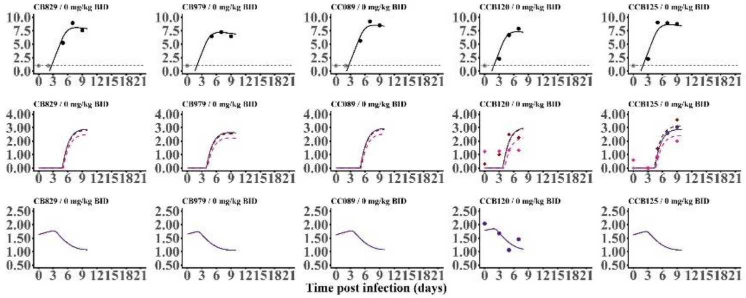
CB829 / 0 mg/kg BID
CB979 / 0 mg/kg BID
CC089 / 0 mg/kg BID
CCB120 / 0 mg/kg BID
CCB125 / 0 mg/kg BID
Time post infection (days)
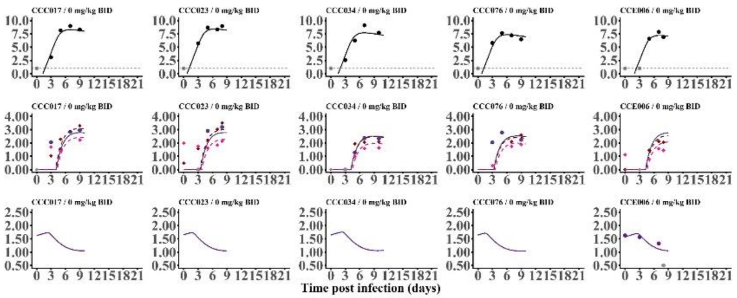
CCC017 / 0 mg/kg BID
CCC023 / 0 mg/kg BID
CCC034 / 0 mg/kg BID
CCC076 / 0 mg/kg BID
CCE006 / 0 mg/kg BID
Time post infection (days)
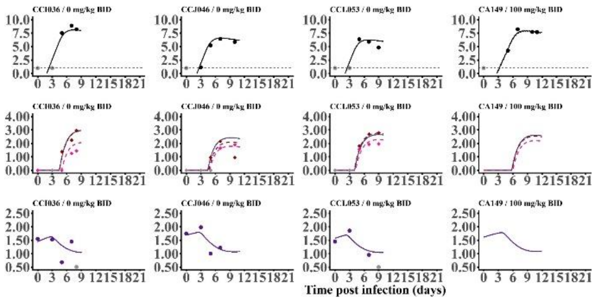
CCI036 / 0 mg/kg BID
CCJ046 / 0 mg/kg BID
CCL053 / 0 mg/kg BID
CA149 / 100 mg/kg BID
Time post infection (days)

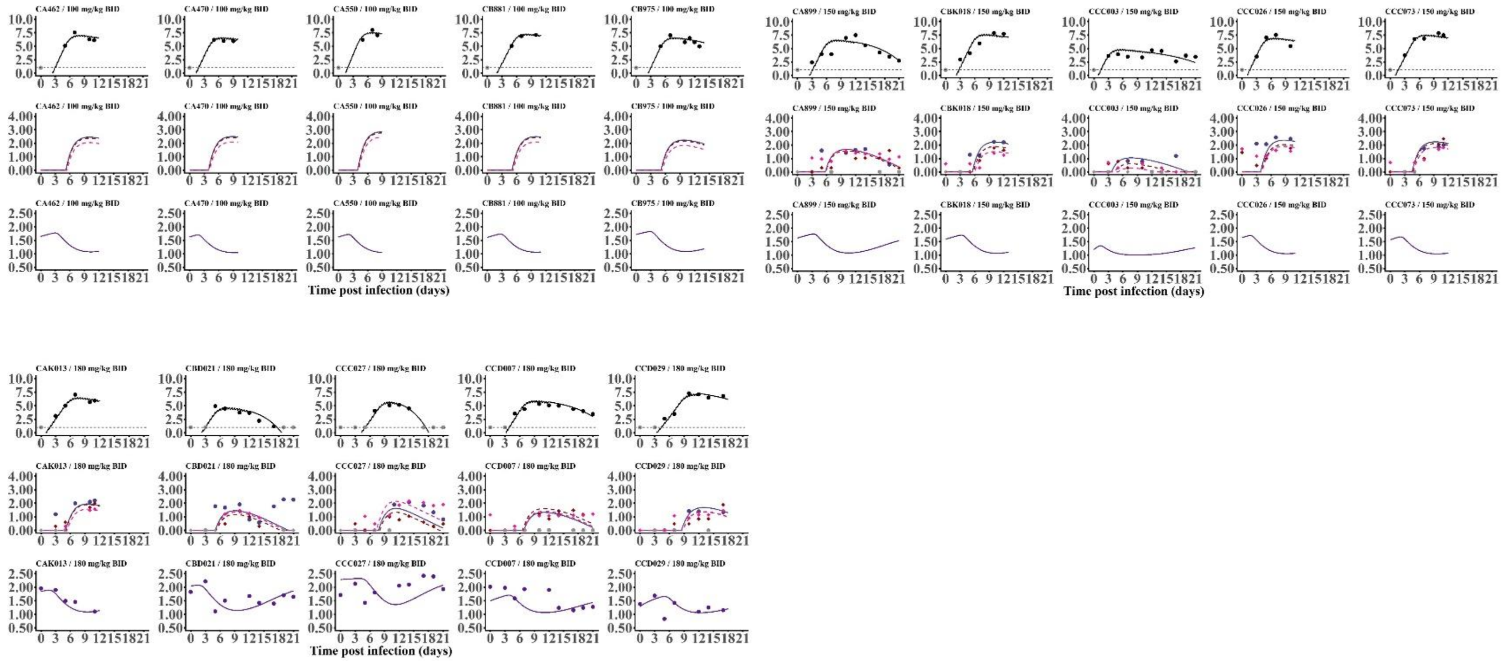

Supplementary Figure 15: Individual fits of integrated variables. Individual fits of molecular viral load (top, black), cytokines (middle, blue: IFNα, red: IL6, pink: TNFα) and CD8 T cells expressing perforin (bottom, purple) in all included animals. Gray dots represent data below the limit of quantitation.

A

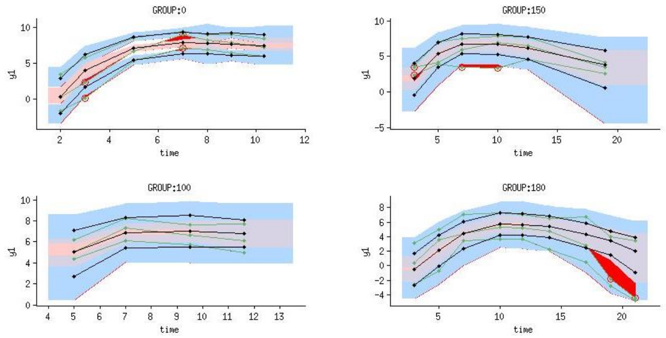

B

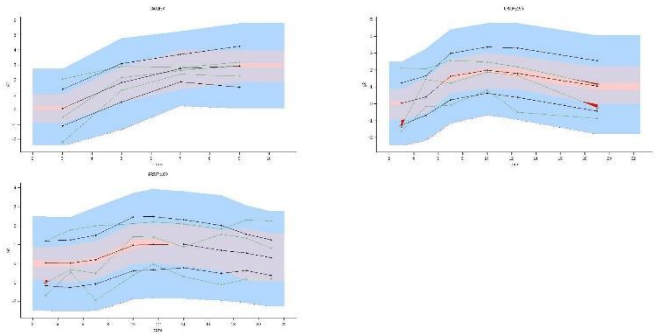

C

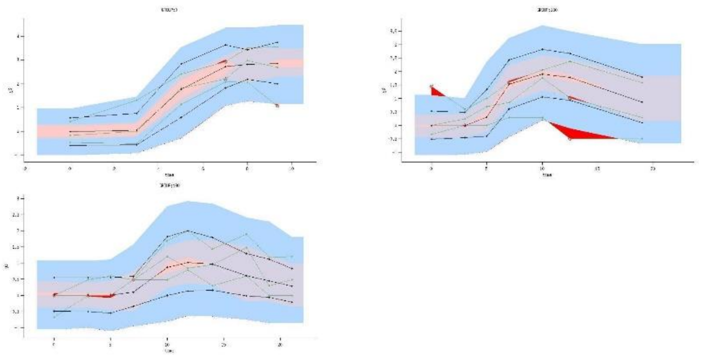

D

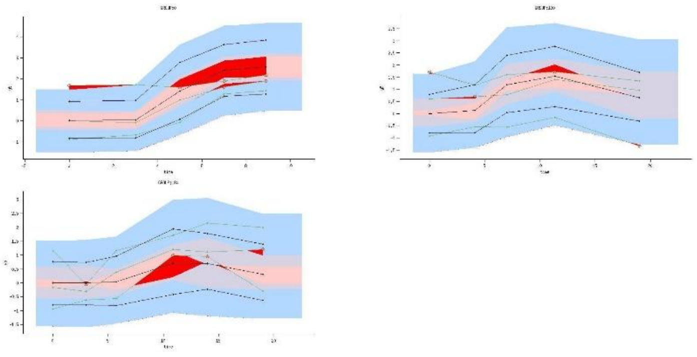

E

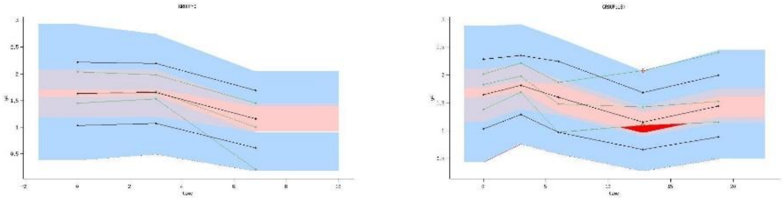

Supplementary Figure 16: Visual predictive checks for the final model stratified per dose groups. a) EBOV viral load, b) IFNα, c) IL6, d) CD8 T cells expressing perforin and e) TNFα. Black lines and dots are the median, 10th and 90th percentiles of model prediction, orange and blue area are their confident intervals. Green lines and dots are the median, 10th and 90th of observations. Red area highlights the observation point which are not included in the confidence interval of the corresponding predicted percentile.

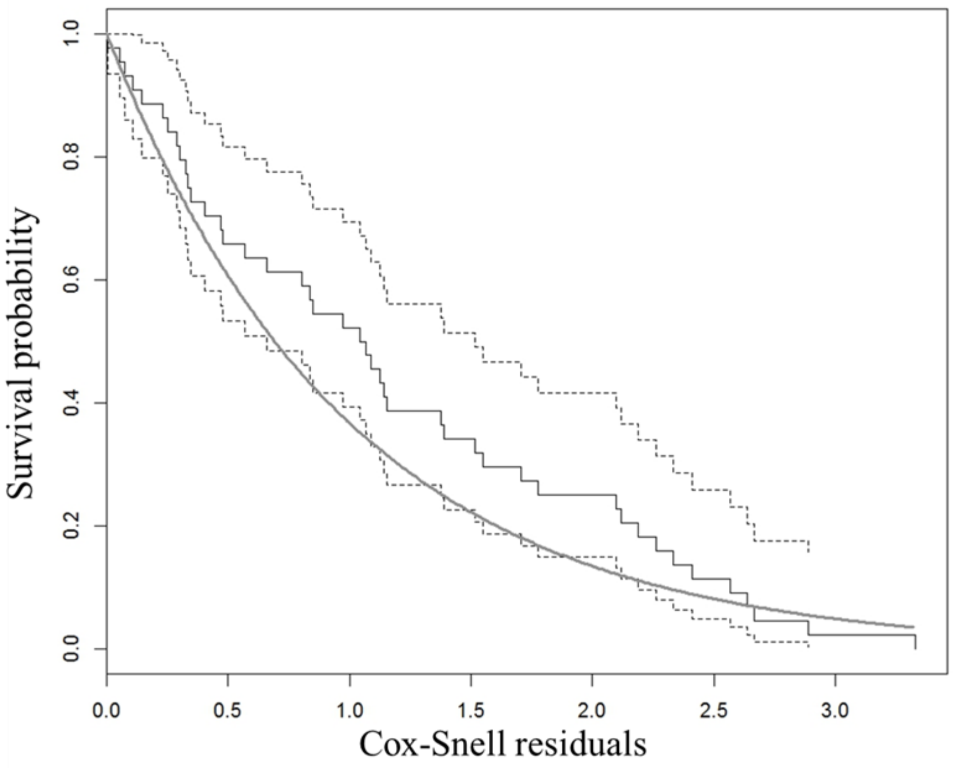

Supplementary Figure 17: Cox Snell residuals of the time to death obtained with the final joint model.

Supplementary Methods

Modeling strategy to characterize favipiravir pharmacokinetics

Experiment conditions in BLS4 laboratory did not allow to perform rich sampling design in the infected animals treated with favipiravir. The pharmacokinetic (PK) model for favipiravir was previously published based on the same dosing regimens in uninfected animals, performed in a low-security laboratory[2]. The data used to construct the model and those collected in the 4 studies performed in the BSL4 (Figure 1) were pooled to 1) evaluate the impact of the Ebola virus disease condition or the laboratory on the pharmacokinetics profile of favipiravir, and 2) to estimate individual concentration profile over time for each animal.

Data:

<u>Data collected in the BSL4 (Sparse data, design in Figure 1):</u>

- Infected animals: PK data in 16 infected animals from experiments 2, 3 and 4 were included: 100 $mg.kg^{-1}$ BID, n=6; 150 $mg.kg^{-1}$ BID, n=5 and 180 $mg.kg^{-1}$ BID, n=5
- Uninfected animals: PK data in 7 animals that were not infected from experiments 2 and 3 were included: 100 $mg.kg^{-1}$ BID, n=3 and 150 $mg.kg^{-1}$ BID, n=4.

<u>Data from previous PK tolerance experiments (Rich data, design in [2]):</u>

- PK concentrations of 30 uninfected animals from previous experiments were included: 60 $mg.kg^{-1}$ BID, n=3; 100 $mg.kg^{-1}$ BID, n= 12; 150 $mg.kg^{-1}$ BID, n=11 and 180 $mg.kg^{-1}$ BID, n=4.

Model:

Favipiravir pharmacokinetics in infected macaques was described using a previously reported pharmacokinetic model of this drug in cynomolgus macaques[2]. Its pharmacokinetics can be described by a one compartment model with two elimination pathways, enzyme dependent and enzyme independent, including an auto-inhibition of favipiravir concentration on the enzyme decreasing over time (Supplementary Equation 1). This model was able to take into account both time dependent and dose dependent non linearity of favipiravir pharmacokinetics [3].

$$\frac{dA_c}{dt} = -k \times A_c - k_{enz} \times A_e \times A_c$$

$$\frac{dA_e}{dt} = R_{in} - k_{out} \times (1 + C_c \times \alpha_{deg} \times e^{-\lambda t}) \times A_e$$

$$R_{in} = k_{out} \times A_{e0}$$

$$C_c = \frac{A_c}{V_d}$$

Supplementary Equation 1

where A_c is the amount of favipiravir in the central compartment, C_c is the favipiravir plasma concentration, A_e is the enzymatic activity level, k is the first-order elimination rate, k_{enz} is the enzyme-dependent first-order elimination rate, k_{out} is the enzyme elimination rate, R_{in} is the zero-order enzyme synthesis rate, α_{deg} is the linear effect of the favipiravir concentration on the enzyme elimination rate, V_d is the volume of distribution and λ the rate at which enzyme elimination becomes less and less sensitive to the favipiravir concentration. The activity level of the enzyme at baseline, $A_e(0)$, was set to 1. This model makes the assumption that favipiravir increases enzyme degradation, in accordance with the irreversible inhibition mechanism proposed by the manufacturer.

Assessment of disease effect on pharmacokinetics and individual predictions:

As the sample design was too sparse to estimate the model parameters in infected macaques, estimation was performed including the data of 30 uninfected cynomolgus macaques (see data and [2]). Effect of experiment center (BSL4/ not BSL4) and of infection were assessed on each model parameter using a forward selection procedure based on BIC.

Then, empirical Bayesian estimates (EBEs) were computed based on the reestimated model parameters and the observed individual trough concentrations to build pharmacokinetic profile of each treated macaque. Plots of observations vs individual predictions and the individual weighted residuals vs time and vs prediction were drawn to ensure the PK model did not introduce bias in the prediction of individual PK exposure.

Parameter estimates in animals from the BSL4 were found to have lower enzyme turnover than the ones from other centers (0.0112 vs 0.0189 day^{-1}, log likelihood ratio test $p < 10^{-6}$). As a result, the macaques handled in the BSL4 experiments had delayed pharmacokinetic peak, occurring about 4 days after treatment initiation, versus 2 days previously (Supplementary

Figure 11). However we could not find any significant effect of the infection on PK parameters, consistent with the trough concentration comparison reported in [4] (Supplementary Table 5).

Plots of observations vs individual predictions (Supplementary Figure 12) and the individual weighted residuals vs time and vs prediction (Supplementary Figure 13) did not point any bias in the individual model predictions, and so in the individual profiles built for each BSL4 animal.

Modeling strategy to characterize the viro-immunological response to EBOV and the impact of treatment

Mechanistic models of acute viral infection were applied to viral and immunological data to characterize physiopathology of the Ebola virus disease leading to fatal outcome, and to assess the impact of antiviral treatment with various levels of effectiveness and timings of initiation.

We here detail all the results related to the modeling strategy and the results provided by alternative models

<u>Modeling strategy</u>

Models of increasing complexity were tested, including successively favipiravir pharmacokinetics, viral load, cytokines, and lymphocytes counts data. Because several cytokines and several lymphocyte markers could be tested, we retained at each step the marker providing the best improvement of viral load data compared to the previous step.

Parameter estimation was performed using nonlinear mixed effect model, allowing to estimate precisely the median parameter values and their between-subject variability. Using this approach, each individual parameters is calculated as the sum of a fixed effect, equal to the median population parameter θ and a random effect, distributed as a Gaussian or Lognormal

distribution of mean 0 and standard deviation ω. The residual error is assumed to be constant for the viral load, cytokines and cytometry measurements on the log scale.

Likelihood maximization was used to estimate parameters using the Stochastic Approximation Expectation Maximization algorithm implemented in Monolix software (http://lixoft.com) to maximize it[5]. The variance-covariance matrix of the parameter estimates was estimated using the inverse of the observed Fisher information matrix (FIM) under the asymptotic assumption and the individual parameters were determined using empirical Bayes estimates.

In order to keep models of increasing complexity comparable, model selection was based on the likelihood of the viral load fitting (the lower the better). This ensures that models not only allow the fitting of cytokine and/or lymphocyte dynamics, but also improve the fitting and hence the understanding of viral load. Random effect selection was performed after the structural model was selected, using a backward procedure based on Bayesian information criterion (BIC).

<u>Models evaluated</u>

1) *Target cell limited model*

The first step was the evaluation of a target cell limited model of acute infection, including an eclipse phase[6] (Supplementary Equation 2). As this model did not incorporate any explicit compartment for immune response, the control of the infection is assumed to result from the depletion of target cells.

$$\frac{dT}{dt} = -\beta TV$$

$$\frac{dI_1}{dt} = \beta TV - kI_1$$

$$\frac{dI_2}{dt} = kI_1 - \delta I_2$$

Supplementary Equation 2

$$\frac{dV}{dt} = p(1-\varepsilon)I_2 - cV$$

$$T_{(t=0)} = T_0;\ I_{1(t=0)} = 0;\ I_{2(t=0)} = 0;\ V_{(t=0)} = V_0$$

Where T are the target cells, I_1 the infected cells in eclipse phase, I_2 the productive infected cells, V the free virions, fitted with the EBOV viral load, β the infectivity constant, k the eclipse phase constant, δ the infected cell elimination rate, p the viral production, T_0 the initial pool of target cells, V_0 is the initial viral load, ε the drug efficacy, function of the plasma drug concentration (cf supra), and c the free virion elimination rate. To take into account the different levels of viral challenge, we modeled V(t=0) = V_0 * inoculum/1000, such that, by construction, the animals infected with 10 or 100 ffu had on average an initial viral load value 100 or 10 fold lower than those infected with 1000 ffu, respectively.

2) *Models including an effect of the innate immune response*

The second step included an explicit compartment for the innate immune response mediated by cytokine levels. Cytokines data were considered on the timeframe of the experiment from D0 to D21 post infection.

Several models were tested (Supplementary Table 6), reflecting the putative effects of the innate response and associated cytokines[7–9]: i) cytokine response enabling target cells conversion into refractory cells ("refractory model", pro-inflammatory cytokines) ii) a deleterious role of the innate response, triggering the production of target cells ("target production model", pro-inflammatory cytokines) iii) cytokine response decreasing viral production ("viral prod inhibition model", pro-inflammatory cytokines) iv) cytotoxic immune response increasing infected cell elimination rate ("cytotoxic model", cytokines related to cellular response).

To support the description of the innate immune compartment, cytokines with compatible biological effect to the biological mechanism were fitted. The five cytokines related to cellular response with significant correlation to survival time at D7 (Supplementary Table 2), namely

IFNγ, IL2, IL15, IL18 and perforin were used to fit the cytotoxic effector compartment of candidate model iv). The three pro inflammatory cytokines with significant correlation to survival time at D7 (Supplementary Table 2), IL6, IFNα and TNFα were used for the innate response compartment of three candidate models i), ii) and iii) assuming inflammation related mechanisms. In order to identify which structural model provides the best description of the data, we performed a selection procedure based on the objective function of the viremia (the lower the better).

In order to be as much consistent as possible and to limit the bias in model selection we assumed in all models that the cytokine production was directly proportional to the number of productive infected cells I_2 such as the cytokine dynamics was given in all models by the following equation:

$$\frac{dF}{dt} = qI_2 - d_F F$$

where q is the apparent cytokine production per infected cell and d_F is the apparent decay rate of cytokines. We therefore ended-up with 14 models to test (Supplementary Figure 14).

Model including an adaptive immune response

The third stage of model building was to evaluate whether the inclusion of cytometry data improved the fit of viral dynamics, in particular the late stage of the infection in the survivor animals. We focused on the three CD8 T cell populations carrying a cytotoxic activity, expressing granzyme B, NKp80 or perforin. A model was used to describe the evolution of these cell populations (Supplementary Equation 3) assuming that each of these populations were made of non-specific CD8 T cells (noted E_1), and EBOV specific CD8 T cells (noted E_2) that eliminated infected cells. In our model, non-specific CD8 T cells decrease after infection, reflecting an inflammation-mediated bystander apoptosis of naïve and memory T cells and/or

indirect apoptosis triggered by EBOV glycoprotein[1011,12]. This loss of non-specific T-cells makes room for the rapid proliferation of specific T-cells.

$$\frac{dT}{dt} = -\beta TV - \frac{\phi TF}{F + \theta_T}$$

$$\frac{dI_1}{dt} = \beta TV - kI_1$$

$$\frac{dI_2}{dt} = kI_1 - \delta I_2 - \kappa I_2 E_2$$

$$\frac{dR}{dt} = \frac{\phi TF}{F + \theta_T}$$

$$\frac{dV}{dt} = p(1-\varepsilon)I_2 - cV$$

$$\frac{dF}{dt} = qI_2 - d_F F$$

$$\frac{dL}{dt} = q_L I_2 - d_L L$$

$$\frac{dN}{dt} = q_N I_2 - d_N N$$

$$\frac{dE_1}{dt} = \sigma - \frac{\zeta F E_1}{F + \theta_E} - \delta_E E_1$$

$$\frac{dE_2}{dt} = \rho E_2\left(1 - \frac{E_2}{E_0}\right) - \delta_E E_2$$

Supplementary Equation 3

$$T_{(t=0)} = T_0;\ I_{1(t=0)} = 0;\ I_{2(t=0)} = 0;\ V_{(t=0)} = V_0;\ F_{(t=0)} = 0;$$

$$L_{(t=0)} = 0;\ N_{(t=0)} = 0;\ E_{1(t=0)} = E_0;\ E_{2(t=0)} = P_0 E_0$$

Where κ is the cytotoxic mediated elimination rate of infected cells, E_0 the baseline count of CD8 T cells, P_0 the initial proportion of specific EBOV CD8 T cells, σ the production rate and δ_E the elimination rate of CD8 T cells, θ_E the cytokine level inducing half of the maximal rate of CD8 T cell elimination and ρ the proliferation rate of specific CD8. *F*, *N* and *L* described

respectively the plasma IFNα, TNFα and IL6 dynamics. All other variables and parameters are identical to Supplementary Equations 2 and Supplementary Table 6.

Modeling the effect of viral and cytokine dynamics on survival

Finally, the fourth step aimed to characterize the survival rate distribution in the different dosing groups. A sequential joint model was built to assess if model prediction of plasma viral load and cytokines could predict survival times, as it was suggested in the descriptive analysis. Population parameters of the longitudinal model selected in the precedent step were fixed to their estimates. We defined *S*(t) the probability to live up time time t (*S*(t)=*P*(T>t), where T is the time of death. *h(t)* was defined as the instantaneous rate of death, depending on the current value of viral load or cytokine predicted by the model: $h(t) = \lambda_m \times \frac{{X_k}^{\gamma}(t)}{{X_k}^{\gamma}(t)+{X_{50}}^{\gamma}}$ or alternatively on the lag-value of viral load or cytokine predicted by the model: $h(t) = \lambda_m \times \frac{{Eff_k}^{\gamma}(t)}{{Eff_k}^{\gamma}(t)+{X_{50}}^{\gamma}}$, where the lag-value was modeled assuming a compartment effect: $\frac{dEff_k}{dt} = k_{eff} \times X_k - k_{eff} \times Eff_k$. In these models λ_m is the maximal hazard in presence of infection, and X_{50} is the current or lag- value of viral load or cytokine inducing hazard value equal to 50% of the maximal hazard, and γ the Hill coefficient. A forward procedure based on Bayesian Information Criterion was used to select the variables to keep in the final model. Then *S*(t) the probability to survive up to time t: $S(t) = e^{-\int_0^t h(u)du}$ was computed from the hazard function.

<u>Parametrization</u>

As done previously[13], evaluated models were reparametrized as a function of the basic reproductive number R_0, which corresponds to the number of new infected cells an productive infected cell will generated during its lifespan. Because some parameters cannot be identified, we fixed them in each evaluated model to plausible values. The free virion elimination rate *c*, was set to 20 day^{-1}, related to other RNA virus half-lives[14]. The initial concentration of target

cell, T_0, was set to 10^8 cells.mL^{-1}, a proxy of the liver size, as hepatocytes were reported to be the largest solid organ targeted by EBOV[15]. The eclipse phase value, k, value is not known, but ranges between 2 and 15 hours[16,17], and was set to 6 hours. Finally the apparent clearance rate of cytokines was fixed to 0.4 day^{-1} by likelihood profiling.

Effect of favipiravir

The individual favipiravir concentration profiles over time obtained from EBEs were injected in the mechanistic viral dynamics model. This two stages approach, with sequential estimation of pharmacokinetics parameters and viral dynamics/treatment effect parameters, was previously described for HCV modeling[18]. Favipiravir is a puric basis analogue[19], with several potential effects hampering the RNA virus replication. The most characterized was the inhibition of the RNA polymerase, it blocks the production of new viral genomes and hence the production of new viral particles[20]. The antiviral effect of favipiravir was therefore modeled as an inhibition of viral production, through an E_{max} model, where the viral production during treatment is written as $p \times (1 - \varepsilon)$, where p is the viral production in absence of treatment, ε the drug efficacy defined as $\varepsilon = \frac{E_{max} \times C}{EC_{50} + C}$, with E_{max} the maximal drug effect, EC_{50} the drug concentration providing 50% of the maximal drug effect and C the drug plasma concentration (see supplementary material 1). Consistent with *in vitro* results and previous model in mice, E_{max} was fixed to 1, assuming that sufficient favipiravir concentration can fully impair viral replication.

Supplementary Note 1

Results of the modeling strategy to characterize the viro-immunological response to EBOV and the impact of treatment

Target cell limited model

The target cell limited model could fit individual data reasonably well but could not capture the dose dependent effect of favipiravir on viremia described in [4]. In particular, it systematically over predicted the viremia observed in the macaques treated with 180 mg.kg^{-1} BID, as shown in Supplementary Figure 15. Estimate of the model parameters were reported in Supplementary Table 7.

Models including an effect of cytokines

Among the four evaluated structural models, the model assuming the conversion of target cells into refractory cells driven by the intensity of the inflammatory response (Supplementary Equation 4) provided the best fit of the EBOV viral load (Supplementary Table 8).

$$\frac{dT}{dt} = -\beta TV - \frac{\phi TF}{F + \theta_T}$$
$$\frac{dI_1}{dt} = \beta TV - kI_1$$
$$\frac{dI_2}{dt} = kI_1 - \delta I_2$$
$$\frac{dR}{dt} = \frac{\phi TF}{F + \theta_T}$$
$$\frac{dV}{dt} = p(1-\varepsilon)I_2 - cV$$
$$\frac{dF}{dt} = qI_2 - d_F F$$
$$\frac{dL}{dt} = q_L I_2 - d_L L$$
$$\frac{dN}{dt} = q_N I_2 - d_N N$$
$$T_{(t=0)} = T_0;\ I_{1(t=0)} = 0;\ I_{2(t=0)} = 0;\ V_{(t=0)} = V_0;$$
$$F_{(t=0)} = 0;\ L_{(t=0)} = 0;\ N_{(t=0)} = 0$$

Supplementary Equation 4

Where *R* are the refractory cells, non-permissive to the infection, *F* the innate immune response compartment, fitted with IFNα plasma concentration, ϕ is the conversion rate, θ_T the cytokine concentration which provide 50% of the maximal conversion rate, *q* is the cytokine production constant, and d_F the cytokine elimination rate. *N* and *L* described respectively the plasma TNFα

and IL6 dynamics, with no effect on the viral replication. All other variables and parameters are identical to Supplementary Equation 2.

A similarly good prediction of the viremia was obtained when assuming that this effect was driven by IFNα, IL6 or TNFα, consistent with the large correlation between these three cytokines. Because the effects of IFNα is supported by *in vitro* experiments[21,22], we decided in the following to include only the effect of IFNα in the model but we kept IL6 and TNFα to stabilize the model and to evaluate their impact on survival. Besides, IFNα production rate q_F estimate of 0.0074 $pg.cell^{-1}.day^{-1}$ was very close to the value reported in previously estimated in another viral infection (murin hepatitis virus, 0.0106 $pg.cell^{-1}.day^{-1}$)[23], and the θ_T estimate was also of same order than the value reported in this publication[23]. As this model assumed that most of the target cells become non permissive to the infection, the extended model with contribution of self-renewal of target cells in their native state was not considered.

Unlike the target cell limited model, this model was able to capture the dose dependent effect of favipiravir on the viremia peak and provided plausible estimates (Supplementary Table 9). However, the declining slope of the viremia, which in this model can be interpreted as the elimination rate of infected cell, was not fully characterized in survivor macaques (Supplementary Figure 16).

Model including an adaptive immune response

Next the model was further extended to evaluate the effect of three CD8 T cell populations (Supplementary Equation 3). Including CD8 T cells expressing granzyme B, NKp80 or perforin improved the description of the EBOV viremia compared to the refractory model, but didi not fully correct the over prediction in the late stage of the disease (Supplementary Figure 17). As

CD8 T cells expressing perforin provided the best objective function, this variable was included (Supplementary Table 10). Parameter estimates were reported in Supplementary Table 11.

Modeling effect of viral and cytokine dynamics on survival

Finally the model was extended to include the survival times of the animals. Models assuming a lag effect of viremia or cytokines with introduction of an effect compartment systematically provided better description of the survival rate than model depending on current marker values (Supplementary Table 12). The best description was provided by a joint model with a hazard of death depending on the lag value of the IFNα concentration or IL6 concentration (Supplementary Table 13). Addition of other variables in the expression of the hazard of death did not improve BIC.

Supplementary References

1. Warren, T. K. *et al.* Therapeutic efficacy of the small molecule GS-5734 against Ebola virus in rhesus monkeys. *Nature* **531,** 381–385 (2016).
2. Madelain, V. *et al.* Favipiravir Pharmacokinetics in Nonhuman Primates and Insights for Future Efficacy Studies of Hemorrhagic Fever Viruses. *Antimicrob. Agents Chemother.* **61,** (2017).
3. Madelain, V. *et al.* Ebola Virus Infection: Review of the Pharmacokinetic and Pharmacodynamic Properties of Drugs Considered for Testing in Human Efficacy Trials. *Clin. Pharmacokinet.* **55,** 907–923 (2016).
4. Guedj, J. *et al.* Antiviral efficacy of favipiravir against Ebola virus in cynomolgus macaques. *2018 Accept. Plos Med.*

5. Lavielle, M. *Mixed Effects Models for the Population Approach: Models, Tasks, Methods and Tools*. (CRC Press, 2014).
6. Baccam, P., Beauchemin, C., Macken, C. A., Hayden, F. G. & Perelson, A. S. Kinetics of influenza A virus infection in humans. *J. Virol.* **80,** 7590–7599 (2006).
7. Pawelek, K. A. *et al.* Modeling within-host dynamics of influenza virus infection including immune responses. *PLoS Comput. Biol.* **8,** e1002588 (2012).
8. Smith, A. M. & Perelson, A. S. Influenza A virus infection kinetics: quantitative data and models. *Wiley Interdiscip. Rev. Syst. Biol. Med.* **3,** 429–445 (2011).
9. Li, Y. & Handel, A. Modeling inoculum dose dependent patterns of acute virus infections. *J. Theor. Biol.* **347,** 63–73 (2014).
10. Jiang, J., Lau, L. L. & Shen, H. Selective depletion of nonspecific T cells during the early stage of immune responses to infection. *J. Immunol. Baltim. Md 1950* **171,** 4352–4358 (2003).
11. Baize, S. *et al.* Defective humoral responses and extensive intravascular apoptosis are associated with fatal outcome in Ebola virus-infected patients. *Nat. Med.* **5,** 423–426 (1999).
12. Geisbert, T. W. *et al.* Apoptosis induced in vitro and in vivo during infection by Ebola and Marburg viruses. *Lab. Investig. J. Tech. Methods Pathol.* **80,** 171–186 (2000).
13. Madelain, V. *et al.* Ebola virus dynamics in mice treated with favipiravir. *Antiviral Res.* **123,** 70–77 (2015).
14. Ramratnam, B. *et al.* Rapid production and clearance of HIV-1 and hepatitis C virus assessed by large volume plasma apheresis. *Lancet Lond. Engl.* **354,** 1782–1785 (1999).
15. Falasca, L. *et al.* Molecular mechanisms of Ebola virus pathogenesis: focus on cell death. *Cell Death Differ.* **22,** 1250–1259 (2015).

16. Towner, J. S. *et al.* Generation of eGFP expressing recombinant Zaire ebolavirus for analysis of early pathogenesis events and high-throughput antiviral drug screening. *Virology* **332,** 20–27 (2005).
17. Hoenen, T., Groseth, A., Callison, J., Takada, A. & Feldmann, H. A novel Ebola virus expressing luciferase allows for rapid and quantitative testing of antivirals. *Antiviral Res.* **99,** 207–213 (2013).
18. Nguyen, T. & Guedj, J. HCV Kinetic Models and Their Implications in Drug Development. *CPT Pharmacomet. Syst. Pharmacol.* **4,** 231–242 (2015).
19. Furuta, Y. *et al.* Favipiravir (T-705), a novel viral RNA polymerase inhibitor. *Antiviral Res.* **100,** 446–454 (2013).
20. Furuta, Y. *et al.* Mechanism of action of T-705 against influenza virus. *Antimicrob. Agents Chemother.* **49,** 981–986 (2005).
21. Stetson, D. B. & Medzhitov, R. Type I interferons in host defense. *Immunity* **25,** 373–381 (2006).
22. Samuel, C. E. Antiviral actions of interferon. Interferon-regulated cellular proteins and their surprisingly selective antiviral activities. *Virology* **183,** 1–11 (1991).
23. Bocharov, G. *et al.* A Systems Immunology Approach to Plasmacytoid Dendritic Cell Function in Cytopathic Virus Infections. *PLOS Pathog.* **6,** e1001017 (2010).
