# Supplementary material for: Ebola viral dynamics in nonhuman primates provides insights into virus immuno-pathogenesis and antiviral strategies
Source: Nat Commun. 2018 Oct 1;9:4013. doi: 10.1038/s41467-018-06215-z (PMC6167368; doi:10.1038/s41467-018-06215-z)
Supplement: Supplementary file 2 — Supplementary Software [file 41467_2018_6215_MOESM2_ESM.docx]

[LONGITUDINAL]

input = {delta,p,c,Tc0, V0, R0, EC50, alphaf, df, F50, beta2, C0, prp, dc, dcv, alphac, lambdac, IFN50,alphail,alphat, delta2,lambdas, betas, keff, Vd, k, Vmax, Kdeg, kel, lambda}

Vd = {use = regressor}

k = {use = regressor}

Vmax = {use = regressor}

Kdeg = {use = regressor}

kel = {use = regressor}

lambda = {use = regressor}

PK:

depot(target=Ac)

EQUATION:

t0=-2

Ac_0 = 0

Ae_0 = 1

Cc = Ac/Vd

tdrug_0=0

ddt_Ac = - k*Ac - Ac*Ae*Vmax

ddt_Ae = Kdeg - Kdeg * (1+Cc*kel*exp(-lambda*tdrug))*Ae

if ( Cc == 0 )

indic=0

else

indic= 1

end

ddt_tdrug= indic

kec=4

Emax=1

Tc_0=10^(Tc0)

I1_0=0

I2_0=0

VI_0=10^(V0)

C_0= C0

Cs_0 = C0/prp

if ( t < 0 )

beta= 0

ct = 0

dct = 0

dcvt = 0

alphact=0

lambdact=0

else

beta= R0*delta*c/p/10^(Tc0);/alphav ;* (1-exp(-kv*t))

ct=c

dct = dc

dcvt = dcv

alphact= alphac

lambdact=lambdac

end

epsilon=Emax*Cc/(Cc+EC50)

ddt_Tc= -beta*VI*Tc -beta2*F*Tc/(F+F50)

ddt_Pc= beta2*F*Tc/(F+F50)

ddt_I1= beta*VI*Tc - kec*I1

ddt_I2= kec*I1 - delta*I2 - delta2*I2*Cs

ddt_VI= p*(1-epsilon)*I2-ct*VI

ddt_F= alphaf*I2 - df*F

ddt_IL= alphail*I2 - df*IL

ddt_C = lambdact - dct*C -dcvt*F/(F+IFN50)*C

ddt_Cs= alphact*Cs*(1-Cs/C0) - dct*Cs

ddt_TNF= alphat*I2 - df*TNF

ddt_EFF= keff*F - keff*EFF

lVL=log10(max(VI,1e-4))

lF=log10(max(F,1e0))

lC=log10(max((C+Cs)/1000,1e-2))

lIL=log10(max(IL,1e0))
