# Supplementary material for: Ebola viral dynamics in nonhuman primates provides insights into virus immuno-pathogenesis and antiviral strategies
Source: Nat Commun. 2018 Oct 1;9:4013. doi: 10.1038/s41467-018-06215-z (PMC6167368; doi:10.1038/s41467-018-06215-z)
Supplement: Supplementary file 4 — Description of Additional Supplementary Files [file 41467_2018_6215_MOESM4_ESM.pdf]

Description of Additional Supplementary Files

File Name: Supplementary Data 1
Description: Original data used in the modeling analysis.

File Name: Supplementary Software
Description: Mlxtran code of the final joint model of favipiravir pharmacokinetics and Ebola virus disease, used in Monolix software to fit the data and estimate model parameter values.
